# Copper‐Induced Transgenerational Plasticity in Plant Defence Boosts Aphid Fitness

**DOI:** 10.1111/pce.15406

**Published:** 2025-01-27

**Authors:** Alexandra Chávez, Anne Schreyer, Pauline Prüsener, Martin Schäfer, Shuqing Xu, Meret Huber

**Affiliations:** ^1^ Institute of Organismic and Molecular Evolution University of Mainz Mainz Rheinland‐Pfalz Germany; ^2^ Institute of Plant Biology and Biotechnology University of Münster Münster Nordrhein‐Westfalen Germany; ^3^ Institute for Evolution and Biodiversity University of Münster Münster Nordrhein‐Westfalen Germany; ^4^ Institute for Quantitative and Computational Biosciences Johannes Gutenberg University of Mainz Mainz Rheinland‐Pfalz Germany

**Keywords:** anthocyanins, aphid herbivory, copper stress, cross‐priming, cross‐resistance, giant duckweed *Spirodela polyrhiza*, jasmonates, mismatched environments, non‐genetic or epigenetic inheritance, transgenerational plasticity

## Abstract

Transgenerational plasticity in plants is an increasingly recognized phenomenon, yet it is mostly unclear whether transgenerational plasticity is relevant to both the fitness of the plant and its interacting species. Using monoclonal strains of the giant duckweed (*Spirodela polyrhiza)* and its native herbivore, the waterlily aphid (*Rhopalosiphum nymphaeae*), we assessed whether pre‐treating plants with copper excess, both indoors and outdoors, induces transgenerational plasticity in plant defences that alter plant and herbivore fitness. Outdoors, copper pre‐treatment tended to increase plant growth rates under recurring copper excess. Indoors, copper pre‐treatment either increased or decreased plant growth rates under recurring conditions, depending on the plant genotype. Copper pre‐treatment induced anthocyanins that protected plants against copper toxicity, and these elevated levels were transgenerationally retained. Copper pre‐treatment also transgenerationally increased the levels of 12‐oxo‐phytodienoic acid (OPDA), a jasmonate precursor. Nevertheless, aphids grew up to 50% better when the plants were pre‐treated with copper. The increased aphid growth was likely caused by transgenerationally elevated OPDA levels, as aphids grew better when jasmonates were externally applied to plants. Taken together, this study shows that transgenerational plasticity is relevant to both plant and herbivore fitness, which highlights the role of transgenerational plasticity in plant evolution and species interactions.

## Summary statement

We show that copper excess can trigger both adaptive and non‐adaptive transgenerational plasticity in the duckweed *Spirodela polyrhiza*. Transgenerational plasticity protected plants when copper excess transgenerationally recurred, but also boosted the growth of the aphid *Rhopalosiphum nymphaeae*.

## 1 Introduction

Since the discovery of Mendel's work, the inheritance of phenotypes across generations has been attributed to the transmission of DNA sequence variation. However, offspring phenotypes may vary also in the absence of DNA sequence change (Bell and Hellmann 2019). Such variation can be due to maternal effects, in which the environment of the mother alters the phenotypes of the direct offspring (Agrawal, Laforsch, and Tollrian 1999). Offspring phenotypes can also be affected by the environment of grandparents (multi‐generational plasticity) or of the great‐grandparents and earlier generations (transgenerational plasticity) (Groot et al. 2016; Huber, Gablenz, and Höfer 2021; Lin et al. 2024). It is, however, largely unclear how common transgenerational plasticity is in plants and whether it affects organismal fitness.

Inferring whether transgenerational plasticity affect organismal fitness is key to assess the evolutionary relevance of this phenomenon. Transgenerational plasticity is not necessarily adaptive: transgenerational plasticity could also be a passive consequence of the previous stress with no or even negative consequences on plant fitness (Baugh and Day 2020; Crisp et al. 2016; Holeski, Jander, and Agrawal 2012; Macartney et al. 2023). Transgenerational plasticity could enhance fitness via at least two non‐exclusive ways: first, induced levels of an adaptive phenotype are retained across generations (‘transgenerationally retained’), which will benefit offspring fitness if the stress recurs. Second, the induced levels bounce back to basal levels in the absence of stress but the adaptive phenotype is induced stronger or faster once the stress recurs in the offspring (‘transgenerationally primed’) (Bell and Hellmann 2019; Holeski, Jander, and Agrawal 2012). Transgenerational plasticity could, however, also lead to the emergence of maladaptive traits (Crisp et al. 2016; Macartney et al. 2023). For example, stress might decrease plant growth, which could reduce resource allocation to offspring and thereby impair offspring fitness (Crisp et al. 2016). As only phenotypes with fitness consequences are evolutionary relevant, it is critical not only to measure phenotypes but also fitness, and to infer the adaptive value of a trait by correlating trait expression to fitness or by manipulating the trait genetically or chemically (Huber et al. 2016). To date, it is largely unclear which transgenerational plastic traits affect organismal fitness.

While most studies in transgenerational plasticity only assessed the consequence on the performance and fitness of the focal plant, transgenerational plasticity may also affect the fitness of interacting species. For instance, *Pieris rapae* larvae gained less weight on *Arabidopsis thaliana* plants whose parents or grand‐parents were exposed to the same herbivore (Rasmann et al. 2012). Furthermore, exposing *A. thaliana* to *Pseudomonas syringae* lowered the performance of the pathogen across two generations (Luna et al. 2011). While these examples highlight that interacting species can be susceptible to multi‐generationally plastic defences that are elicited by an initial biotic stress, it is unclear whether interacting species are also sensitive to multi‐generationally plastic defences that are induced by an abiotic stress. As different stresses can induce shared but also antagonistic signalling and defence pathways (Chang et al. 2020; Gallusci et al. 2023), exposure to one stress may prime the response of the offspring within another stress, thus causing cross‐priming in mismatched environments, and leading to cross‐resistance when the priming is beneficial (Lämke and Bäurle 2017; Liu, Able, and Able 2022). Considering that abiotic stresses are prevalent in nature and that herbivores and pathogens often invade plant populations in irregular intervals, studying whether transgenerational plasticity induced by an abiotic stress can alter the fitness of interacting species may reveal unexpected yet common processes in nature. Furthermore, such transgenerational cross‐priming could be exploited to generate plants with increased resistance in the field (Vázquez‐Hernández et al. 2019; Villagómez Aranda et al. 2022; Yang, Zhi, and Chang 2022).

To infer that transgenerational plasticity is relevant for organismal fitness, the first step is to demonstrate that the traits is truly transgenerationally plastic. However, these demonstrations are rare, as several requirements must be met: first, the phenotypic effects must be independent of DNA sequence variation. DNA sequence variation is minimal in monoclonal or highly inbred lineages, especially in species with low mutation rates. Second, selection of any pre‐existing or spontaneously arising genetic and epigenetic variation must be avoided. This can be achieved using single descendant lineages, in which each generation is founded by a single, randomly selected descendant, thereby almost completely avoiding the effect of selection (Baugh and Day 2020). Third, the offspring should not have been exposed to the initial stress: as mother plants already contain the cells that will form the next generation, effects in the first generation can be caused by direct stress exposure, and thus effects in the second generation can be maternal effects (Grossniklaus et al. 2013). Consequently, only effects in the third generation are not confounded by direct stress exposure or maternal effects and are transgenerationally plastic. Fourth, to conclude that a trait is transgenerationally plastic because some epigenetic or other plant‐derived factors are inherited, one should perform the experiments under axenic conditions. Otherwise, the initial stress may alter the composition of plant‐associated microbes, which may be vertically transmitted and could affect offspring phenotypes (Baldassarre et al. 2022; Gundel, Rudgers, and Whitney 2017).

Transgenerational plasticity seems to be rare in plants reproducing sexually (Bell and Hellmann 2019; Groot et al. 2016; Lin et al. 2024; Sánchez‐Tójar et al. 2020; Suter and Widmer 2013), possibly because environment‐induced epigenetic marks—which could alter gene expression and phenotypes (Cubas, Vincent, and Coen 1999)—can be erased during meiosis (Crevillén et al. 2014; Iwasaki and Paszkowski 2014; Ono and Kinoshita 2021; Tao et al. 2017). Transgenerational plasticity appears to be more common in asexually reproducing plants, possibly because epigenetic marks can be inherited faithfully through mitosis (He and Li 2018). Evidence of transgenerational plasticity in asexual plants is nevertheless scarce (Huber, Gablenz, and Höfer 2021; Van Antro et al. 2023), particularly under natural conditions outdoors, possibly because transgenerational plasticity has been studied less frequently in asexual than in sexually reproducing plants (Macartney et al. 2023). As most plants can reproduce asexually (Hutchinson 1975; Klimeš et al. 1997; Pyšek 1997; Tiffney and Niklas 1985), and as asexually reproducing plants include many crops, invasive weeds and keystone species (McKey et al. 2010; Pyšek 1997), transgenerational plasticity in asexual plants may play an underappreciated role in agriculture, ecosystem functioning and species evolution.

Two asexually reproducing species that are particularly suitable to study whether traits are transgenerationally plastic and fitness‐relevant are the giant duckweed (*Spirodela polyrhiza)* and its native herbivore, the waterlily aphid (*Rhopalosiphum nymphaeae*). *S. polyrhiza* reproduces rapidly and almost exclusively asexually by budding; under ideal conditions, the plant produces a new vegetative generation approximately every 2 days (Ziegler et al. 2015). *S. polyrhiza* has a simple body structure with a flat, thallus‐like shoot (‘frond’), which allows to precisely estimate fitness across several generations by measuring the increase in frond surface area. *S. polyrhiza* often grows in small water bodies near human‐influenced environments, and these water bodies often contain fluctuating levels of anthropogenically derived chemicals such as copper, a major pollutant in aquatic ecosystems (Ruas, Costa, and Bered 2022). *S. polyrhiza* defends against these stresses likely through the production of antioxidative flavonoids, including several flavones and two major anthocyanins, cyanidin‐3‐malonylglucoside and cyanidin‐3‐glucoside (Böttner et al. 2021; Liu, Li, et al. 2022). In nature, *S. polyrhiza* is attacked by *R. nymphaeae*, a generalist aphid that, in summer, switches from terrestrial hosts to several aquatic hosts including duckweeds (Center et al. 2002). Under favourable conditions, *R. nymphaeae* reproduces parthenogenetically on duckweeds, giving live birth to a new generation approximately every 6 days (Hance et al. 1994). The rapid asexual reproduction of both *S. polyrhiza* and *R. nymphaeae* allows to assess the consequence of transgenerational plasticity on both plant and herbivore fitness.

In this study, we tested whether exposing *S. polyrhiza* to copper excess outdoors and indoors triggers transgenerational plasticity in plant defences that alter plant and herbivore fitness. By measuring plant and herbivore fitness, and manipulating transgenerationally plastic plant defences, we show that copper‐induced transgenerational plasticity can have unexpected consequences on the fitness of interacting species, highlighting the role of transgenerational inheritance in plant evolution and species interactions.

## 2 Materials and Methods

### 2.1 Plant Material and Growth Conditions

We used six different *S. polyrhiza* genotypes with cosmopolitan distribution (Supporting Information: Table S1), covering three of the four genetic clusters (Wang et al. 2024; Xu et al. 2019). Indoors, plants were cultivated inside growth cabinets (GroBank, CLF PlantClimatics, Wertingen, Germany) operating at either 26°C and 135 µmol photons m^2^ s^−1^ for the outdoor experiment, or 28°C and 150 µmol photons m^2^ s^−1^ for the indoor experiments, both within a 16:8 h light/dark cycle, and using the optimal growth‐supporting N‐medium (Appenroth, Teller, and Horn 1996). To measure *S. polyrhiza* growth rates, we took a picture of the plants at the beginning and end of assays using a camera box installation with a webcam (HD Pro Webcam C270, Logitech, Lausanne, Switzerland; webcam software 2.12.8).

### 2.2 Statistical Analysis

All data were analysed in R v4.4.0 (R Core Team 2024). As fitness parameter, we calculated relative growth rates, here called ‘growth rates’, based on *S. polyrhiza* surface area, as well as *R. nymphaeae* number. Growth rates are the difference between the natural logarithm of the final and initial values, divided by the days the plants or aphids grew (Hunt 1982) (Supporting Information: Equation S1). To estimate surface area, we used ImageJ 64 v5 (Schneider, Rasband, and Eliceiri 2012). Indoors, we estimated as morphology traits: surface area per frond (mm^2^), fresh weight per frond (mg FW) and surface area per fresh weight (mm^2^ mg^−1^ FW). To count the final fronds in the assays we used dotdotGoose v1.5.3 (Ersts). Additionally, we estimated the pre‐treatment ratio of the different variables as the rate between the copper pre‐treated plants relative to the mean value of the control pre‐treated plants within each treatment environment (Huber, Gablenz, and Höfer 2021) (Supporting Information : Equation S2).

Mixed effects models were performed with the package glmmTMB v1.1.9 (Brooks et al. 2017) for all analyses, except for the screening of altered metabolites per environment, conducted with lme4 v1.1.34 (Bates et al. 2015). *p*‐values of the fixed‐effects models were estimated with analysis of variance (ANOVA), using Wald chi‐square of the package car (R Core Team 2024). *R* ^2^ values were obtained with ggpmisc v0.5.6 (Aphalo 2024). Post‐hoc *p*‐values were calculated with emmeans v1.10.2 (Lenth 2024) and letters were obtained with multcomp v1.4.25 (Hothorn, Bretz, and Westfall 2008). We used the package DHARMa v0.4.6 (Hartig 2022) to verify whether the scaled residuals of our fitted models correlated with the simulated values from the same model, and the package effects v4.2.2 (Fox and Weisberg 2018) to extract fit values. To estimate deviation from neutral, we performed Wilcoxon Rank‐Sum tests of the pre‐treatment ratios per environment against one (mu = 1). We displayed plots with ggplot2 v3.4.3 (Wickham 2016) and organized data with readxl v1.4.2 (Wickham and Bryan 2023), data.table v1.14.8 (Barrett et al. 2024), tidytext v0.4.1 (Silge and Robinson 2016) and dplyr v1.1.2 (Wickham et al. 2023).

### 2.3 Effects of Copper Pre‐Treatment on Plant Fitness Outdoors

**Figure 1:**
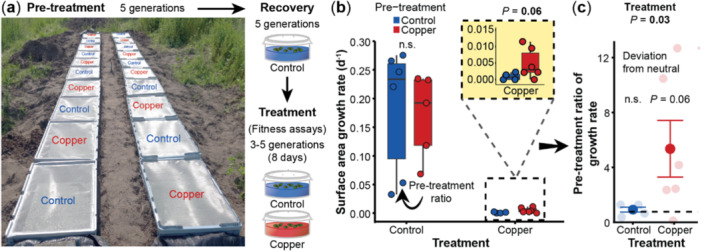
Copper excess outdoors benefits *Spirodela polyrhiza* fitness under recurrent copper stress. (a) Overview of the outdoor experimental setup. Monoclonal *S. polyrhiza* plants of genotype SP004 were grown for five generations as single descendants outdoors in the presence and absence of copper excess, and after five generations of recovery indoors, plant fitness and phenotypes were assessed after 8 days of free growth in each environment. (b) Copper pre‐treatment tended to increase surface area growth rates upon recurring copper excess. *p*‐values above each boxplot pair refer to mixed effects models per treatment. (c) The pre‐treatment ratios of surface area growth rates (growth rates of copper pre‐treated plants relative to the mean growth rate of control pre‐treated plants) were higher under copper than control conditions. Bold circles represent the mean value and error bars the standard error. Background circles represent individual pre‐treatment ratio values. Statistical analysis on the deviations from neutral (horizontal dotted line) refer to Wilcoxon Rank‐Sum tests. Variation between treatments corresponds to a linear regression. *N* = 4–6.

To test whether copper excess under natural conditions alters *S. polyrhiza* fitness under transgenerationally recurring copper excess, we grew genetically uniform *S. polyrhiza* populations of genotype SP004 (Clone 7498, Supporting Information: Table S1) outdoors in the presence and absence of copper excess (Figure 1a). The genotype was chosen because it previously displayed transgenerational plasticity upon copper excess (Huber, Gablenz, and Höfer 2021). Within the populations, we followed monoclonal, single descendant lineages for five generations (‘pre‐treatment’, Supporting Information: Figure S1) and after five additional generations of growth in the absence of stress, we assessed *S. polyrhiza* fitness in the presence and absence of recurring copper excess.

#### 2.3.1 Pre‐Treatment Phase

In May 2021, we set up 20 ponds (80 × 60 × 32 cm, AuerPackaging, Amerang, Germany) inside an open field site in Münster, Germany (51°57′54.0′N, 7°36′22.4′E). Ponds were buried to the rim with soil and filled with 10 L of pond soil (Floragard, Oldenburg, Germany), 40 mL of organic fruit and vegetable fertilizer (organic NPK 3.1 + 0.5 + 4.1; COMPO, Münster, Germany) and tap water with a total volume of 100 L. The boxes had a draining scape and lids consisting of a stainless‐steel mesh (mesh opening 0.63 mm, wire diameter, 0.224 mm; Haver&Boeker, Oelde, Germany). On May 26th, each box received around 3000 *S. polyrhiza* fronds of the genotype SP004 that had been pre‐cultivated indoors for 1 month in 10% N‐medium at 26°C, 135 µmol photons m^−2^ s^−1^ and 16:8 h light/dark cycle. After 1 week of acclimation, we added 20 µM CuSO_4_ to half of the ponds (*N* = 10) to initiate the stress pre‐treatment (Figure 1a). This CuSO_4_ concentration reduced plant growth indoors by approximately 40% (Huber, Gablenz, and Höfer 2021). Across the entire experiment, we measured copper concentration weekly and after each rainfall using the HI702 Checker Copper HS (Hanna instruments, Vöhringen, Germany); if needed, we added CuSO_4_ to maintain 20 µM CuSO_4._ After adding CuSO_4_ for the first time, we randomly chose four plants per pond as the founders of the single descendant lineages. To trace the single descendant lineages, we marked the plants with a dot using a permanent marker (Stabilo OHPen Universal, Heroldsberg, Germany) (Supporting Information: Figure S1). The remaining population reproduced freely and covered the surface after 12 days, after which we randomly removed 30% of the plants to avoid crowding. We grew the single descendants within the population to simulate natural conditions, as in nature *S. polyrhiza* usually grows in populations rather than as isolated plants, and isolated plants often stop reproducing because of competing algae (Böttner et al. 2024).

#### 2.3.2 Recovery Phase

After five generations of pre‐treatment, we moved the sixth generation of the single descendants (approx. 24 days of pre‐treatment) into separate cavities of polyvinyl chloride disks (7 cm diameter) that were floating inside the control ponds. Due to algae bloom, these plants did not produce any offspring outdoors. We thus transferred the very same plants to the initial indoor conditions (after 38 days of outdoor conditions), within 250 mL polypropylene beakers (Plastikbecher GmbH, Giengen an der Brenz, Germany) filled with 150 mL 10% N‐medium and covered with perforated transparent lids. The lineages of each pond were kept in separate beakers. To reduce algae growth, we changed the medium every 2–3 days and wrapped the sides of the beakers with three layers of black plastic foil to decrease light incidence from the sides. Inside the plastic beakers, we propagated each lineage as singles descendants for five generations (‘recovery’).

#### 2.3.3 Fitness Assays

To assess *S. polyrhiza* fitness under recurring copper excess, we subsequently placed either the first or the second offspring into separate 250 mL polypropylene beakers containing control conditions (10% N‐medium) and copper excess (20 µM CuSO_4_) (‘treatment’), with half of the replicates per treatment using the first offspring, and half of the replicates the second offspring. Subsequently, the plants grew freely for 8 days. Towards the end of the assays, plants died under recurrent stress, likely because outdoor grown plants were weakened and could not resist otherwise sublethal stress levels. We nevertheless took a picture to assess growth rates.

To test whether copper pre‐treatment alters *S. polyrhiza* growth rates, we fitted mixed effects models using as random factor the pair of control and copper ponds (‘Pair’): Growth ~Pre‐treatment*Treatment + (1|Pair). Additionally, we analysed the pre‐treatment effect within each treatment using the model Growth ~Pre‐treatment + (1|Pair). To assess the effect of the treatment on the pre‐treatment ratio (i.e., the trait value of copper pre‐treated plants relative to the mean trait value of the control pre‐treated plants in the respective environment), we used linear models (Pre‐treatment ratio ~Treatment).

### 2.4 Effects of Copper Pre‐Treatment on Plant and Aphid Fitness and Phenotype Indoors

**Figure 2:**
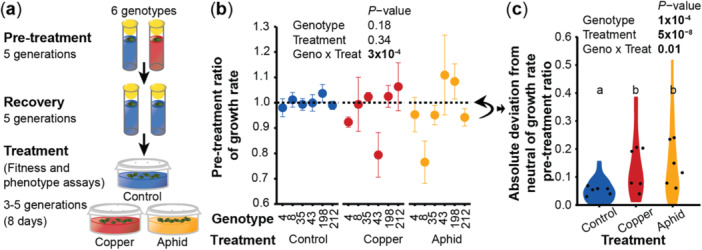
Copper excess indoors alters *Spirodela polyrhiza* fitness in a genotype‐ and environment‐dependent manner. (a) Overview of the indoor experiment. Monoclonal *S. polyrhiza* of six different genotypes were grown as single descendants for five generations indoors in the presence and absence of copper excess, and after five generations of recovery under control conditions, plants fitness and phenotypes were assessed after 8 days of free growth under control conditions, copper excess and aphid herbivory. (b) The pre‐treatment ratios of surface area growth rates (growth rates of copper pre‐treated plants relative to the mean growth rate of control pre‐treated plants) depended on the interaction of the genotype and the treatment. Circles represent the mean value and error bars the standard error. *p*‐values refer to a mixed effects model. *N* = 3–6 per genotype (c) Copper pre‐treatment ratios of growth rates deviated more strongly from neutral under copper excess and aphid herbivory than under control conditions. Black dots represent the mean value per genotype within a treatment. *p*‐values refer to a mixed effects model. Pairwise comparisons were obtained with least‐squares means performed on treatments from a mixed effects model. *N* = 28–32. Geno, genotype; Treat, treatment. [Color figure can be viewed at wileyonlinelibrary.com]

To assess whether copper pre‐treatment alters *S. polyrhiza* fitness and phenotype in different genotypes under axenic conditions, and whether copper pre‐treatment alters also herbivore fitness, we pre‐treated single descendant lineages of six *S. polyrhiza* genotypes—including the genotype used outdoors (Supporting Information: Table S1)—for five generations with and without copper excess indoors, followed by five generations of recovery under control conditions and finally 8 days of fitness and phenotype assay under control conditions, copper excess or herbivory of the aphid *R. nymphaeae* (Figure 2a).

#### 2.4.1 Pre‐Treatment Phase

To acclimate the plants prior to the experiment, we grew six surface‐sterilized plants per genotype for four generations as single descendants inside 30 mL polypropylene tubes (Fisher Scientific, Waltham, USA) filled with 25 mL N‐medium and closed with sterilized foam plugs (CarlRoth, Karlsruhe, Germany). The first and second offspring of the fourth generation were placed inside new polypropylene tubes filled with N‐medium with and without copper excess (20 µM CuSO_4_), with half of the replicates per pre‐treatment coming from the first offspring and half from the second offspring (Supporting Information: Figure S2.1). Plants were grown as single descendants under these conditions for five generations (‘pre‐treatment’).

#### 2.4.2 Recovery Phase and Fitness and Phenotype Assays

After five generations of pre‐treatment, we grew the plants inside the polypropylene tubes for another five generations under control conditions (‘recovery’). Subsequently, to assess the effect of copper pre‐treatment on plant fitness and phenotype, we placed the next generations into 250 mL transparent polypropylene beakers filled with 150 mL N‐medium and covered with perforated transparent lids, following a structured branching design that harnesses enough plants without introducing biases in the offspring sequence (Supporting Information: Figure S2.1). We allowed the plants to grow freely for 8 days under three treatments: control conditions, copper excess (20 µM CuSO_4_), and aphid herbivory (three adult aphids per frond) (‘treatment’, Figure 2a). Subsequently, we measured *S. polyrhiza* growth rates as mentioned above, counted the number of aphids, and harvested plants by briefly drying them with a tissue paper, weighting them and subsequently flash‐freezing them in liquid nitrogen. Samples were stored at ‐80°C until metabolite extraction. Because the aphids contaminated some replicates with algae, we excluded those replicates from the analysis. The experiment was carried out under axenic conditions, except during the 8 days of fitness and phenotype assays within the treatment phase.

#### 2.4.3 Effects of Copper Pre‐Treatment on *Spirodela polyrhiza* Fitness and Morphology

To assess the effect of the pre‐treatment on *S. polyrhiza* fitness and morphology of all six genotypes, we used mixed effects models considering as random factors the replicate from which the pre‐treated and treated samples derived (‘Replicate’), the developmental pockets observed when starting assays (‘Offspring’), the rack where plants were propagated during the experiment (‘Rack’) and the offspring used at the beginning of assays (‘Branching’) (Supporting Information: Figure S2.1). Details on the models are described in Supporting Information: Methods S1.

#### 2.4.4 Effects of Copper Pre‐Treatment on *Rhopalosiphum nymphaeae* Fitness

To identify the effects of copper pre‐treatment on growth rates of *R. nymphaeae*, we used the mixed effects model Growth ~Pre‐treatment + (1|Replicate) + (1|Genotype) + (1|Branching), including the replicates of all six *S. polyrhiza* genotypes. Additionally, to estimate the effect of these genotypes, we used the model Growth ~Pre‐treatment*Genotype + (1|Replicate) + (1|Branching). To assess the effects of the six *S. polyrhiza* genotypes on *R. nymphaeae* growth rate pre‐treatment ratios, we used the model Pre‐treatment ratio ~Genotype + (1|Branching). To correlate the pre‐treatment ratios of both *S. polyrhiza* fitness and morphology to the pre‐treatment ratio of aphid fitness, we used the model Pre‐treatment ratios of duckweed ~Pre‐treatment ratios of aphid + (1|Genotype) + (1|Branching), considering the replicates of all six *S. polyrhiza* genotypes.

#### 2.4.5 Effects of Copper Pre‐Treatment on *Spirodela polyrhiza* Metabolites

To identify whether copper pre‐treatment alters the concentrations of metabolites upon recurring stresses, we screened the levels of phytohormones, free amino acids, amines and specialized metabolites of the phenylpropanoid pathway using all replicates from the six genotypes collected in the fitness and phenotype assays (Supporting Information: Table S1). Thereto, we used the target screening method of Schäfer et al. (2016), with the modifications described in Malacrinò et al. (2024). In short, we applied into the samples acidified methanol (MeOH:water:formic acid 15:4:1 v/v/v) containing the internal standards for the phytohormones and phenylpropanoid quantification. An aliquot of the extract was set apart to quantify flavonoids and chlorogenic acid. To quantify free amino acids, another aliquot of the extract was diluted 1:100 in an aqueous mix of isotope‐labelled amino acids (algal amino acid mixture‐13C‐15N; Sigma‐Aldrich). Finally, phytohormones and several phenylpropanoids were purified and partially concentrated from the remaining extract through two solid‐phase extraction steps utilising Chromabond HR‐X and HR‐XC columns (Macherey‐Nagel, Düren, Germany).

To quantify flavonoids and the chlorogenic acid, we used the Nexera XR HPLC System (Shimadzu, Duisburg, Germany) which was equipped with a 5 µM EC 4/3 Nucleodur Sphinx RP pre‐column (Macherey‐Nagel), a 250 × 4.6 mm 5 µM Nucleodur Sphinx RP column (Macherey‐Nagel) and a PDA detector (Shimadzu). The mobile phase comprised of an aqueous solution of 0.2% formic acid (Fisher Scientific) with 0.1% acetonitrile (Fisher Scientific) as solvent A and acetonitrile as solvent B in gradient mode. We used the gradient programs, the column oven settings for the chromatographic separation, and the detector settings described in Malacrinò et al. (2024). The absolute concentrations of the flavones, anthocyanins and chlorogenic acid were calculated based on external standard curves of identical compounds, except for cyanidin‐3‐malonylglucoside that was quantified based on the cyanidin‐3‐glucoside standard.

The analysis of amino acids, amines, and phytohormones was performed on a Nexera X3 UHPLC‐System (Shimadzu) connected to a LCMS 8060 mass spectrometer (Shimadzu). The LC‐System was equipped with a 0.3 µM 1290 infinity II pre‐column (Agilent Technologies, Frankfurt am Main, Germany) and a 50 × 3 mm, 1.83 µM Zorbax Eclipse XDB‐C18 column (Agilent Technologies, Frankfurt am Main, Germany). The mobile phase comprised of an aqueous solution of 0.05% formic acid with 0.1% acetonitrile, as solvent A and methanol (Fisher Scientific) as solvent B in gradient mode. The mass spectrometer was equipped with an electrospray ionization (ESI) source and operated in multi‐reaction monitoring (MRM) modus. The MRM‐settings, ESI‐settings, the gradient program and column oven settings were used as described in Malacrinò et al. (2024), with the addition of a putative chlorogenic acid isomer to method 1A (Supporting Information: Table S2). Analytes were quantified based on internal standards as described in Malacrinò et al. (2024) and Supporting Information: Table S2. The peaks from all metabolites were integrated with LabSolution Version 5.123 (Shimadzu, Duisburg, Germany) for the HPLC‐PDA data and with LabSolution Insight Version 4.0 SP6 (Shimadzu) for the LC‐MS/MS data.

To screen for metabolites that were affected by the pre‐treatment within each treatment, we used mixed effects models, considering as additional random factors the plate used for the extraction (‘Plate’) and the batch of samples extracted and read at one time by the machines (‘Batch’). Thereto, we used the model Concentration ~Pre‐treatment + (1|Genotype) + (1|Replicate) + (1|Plate) + (1|Batch), with slight variations depending on the specific questions (Supporting Information: Methods S2). All metabolite analyses included the replicates of all six genotypes.

#### 2.4.6 Benefits of Transgenerationally Plastic Metabolites Under Stress

To infer whether anthocyanins and tryptamine—which were transgenerationally retained—protect plants against copper excess and aphid herbivory, we correlated the concentrations of these metabolites with *S. polyrhiza* fitness in each environment using the control pre‐treated plants of all genotypes. Thereto, we used the model Pre‐treatment ratio of growth rates ~pre‐treatment ratio of metabolites + (1|Genotype) + (1|Replicate), considering the replicates of all six genotypes.

To test whether the level of 12‐oxo‐phytodienoic acid (OPDA)—which was transgenerationally plastic—were positively correlated with the levels of the defence hormones jasmonic acid and jasmonic acid‐isoleucine, we used the model Pre‐treatment ratios of jasmonates ~pre‐treatment ratios of OPDA + (1|Genotype), considering the replicates of all six genotypes.

### 2.5 External Application of Methyl Jasmonate on Plants

To assess whether transgenerational plasticity in OPDA affects *R. nymphaeae* fitness, we externally applied methyl jasmonate to *S. polyrhiza* and subsequently assessed *R. nymphaeae* growth rates. Thereto, we placed three individual fronds of genotype SP050 (Supporting Information: Table S1), a standard genotypes in our laboratory, into 50 mL falcon tubes filled with 3 mL of either N‐medium or 100 µM methyl jasmonate in N‐medium (‘Induction’, *N* = 12 per treatment). To effectively dissolve the methyl jasmonate, 0.5% DMSO was added to both the control and methyl jasmonate‐containing N‐medium. We split the experiment into three batches, performed one after the other. After 72 h in the presence and absence of methyl jasmonate, each colony formed from the three fronds was moved into individual 250 mL transparent polypropylene beakers filled with 150 mL N‐medium and subjected to either control conditions or aphid herbivory (three adult aphids per plant). After 2 days, we counted the number of aphids. The experiment was repeated once in a similar way to confirm the findings and to assess whether methyl jasmonate induces OPDA, jasmonic acid and jasmonic acid‐isoleucine (Supporting Information: Methods S3).

To assess whether externally applied jasmonates alter *R. nymphaeae* growth rates, we used mixed effects models considering as an additional random factor the batch of samples worked per day (‘Group’). The model was Growth ~Induction*Treatment + (1|Replicate) + (1|Group) (Supporting Information: Methods S4). Two samples per treatment were excluded from the growth rate analysis as the aphids did not produce any offspring.

## 3 Results

### 3.1 Copper Pre‐Treatment Outdoors Tends to Benefit Plant Fitness Under Transgenerationally Recurring Copper Stress

To assess whether copper excess alters *S. polyrhiza* fitness under transgenerationally recurring copper excess in natural conditions, we pre‐treated plants with copper excess outdoors, and after five generations of growth under control conditions, we assessed plant fitness in the presence and absence of recurring copper excess (Figure 1a). While copper pre‐treatment did not affect *S. polyrhiza* growth rates under control conditions, copper pre‐treatment tended to increase plant growth rates by approximately 400% under copper excess (*p* = 0.06, mixed effects model, Figure 1b, Supporting Information: Table S3). Similarly, the pre‐treatment ratios, i.e. the ratio between plant growth rates of copper compared to control pre‐treated plants, differed between control and copper conditions (*p* = 0.03, mixed effects model, Figure 1c): under control conditions, copper pre‐treatment ratios were close to neutral, whereas under copper excess, the pre‐treatment ratios tended to be positive (deviation from neutral in control: *p* > 0.1; in copper: *p* = 0.06, Wilcoxon rank sum test, Figure 1c).

### 3.2 Copper Pre‐Treatment Indoors Alters Plant Fitness and Morphology in an Environment‐ and Genotype Dependent Manner

To assess whether copper pre‐treatment alters *S. polyrhiza* fitness not only under copper excess but also under herbivory of the waterlily aphid *R. nymphaeae*, and to test whether these transgenerational effects are genotype‐specific, we grew six world‐wide sampled genotypes (Supporting Information: Table S1) for five generations as single descendants indoors under control and copper excess, followed by five generations under control conditions prior to fitness assays under control conditions, copper excess and aphid herbivory (Figure 2a). While copper pre‐treatment did not affect plant surface area growth rates when expressed in raw values (Supporting Information: Figure S2.2), copper pre‐treatment altered the growth rates in a genotype and environment‐dependent manner when expressed as pre‐treatment ratios (*p* [genotype × treatment] = 3 × 10^−4^, mixed effects model, Figure 2b): under control conditions, copper pre‐treatment did not affect *S. polyrhiza* growth rates, whereas under copper excess and aphid herbivory, copper pre‐treatment either increased or decreased *S. polyrhiza* growth rates, depending on the genotype. These pre‐treatment effects were not marginal: in some genotypes, copper pre‐treatment reduced *S. polyrhiza* growth rates by up to 25%, whereas in other genotypes, copper‐pretreatment increased growth rates by 10%. Across all genotypes, growth rate pre‐treatment ratios were close to neutral under control conditions but deviated from neutral, both positively and negatively, under copper excess and aphid herbivory (absolute deviation from neutral among treatments: *p* = 5 × 10^−8^, mixed effects model, Figure 2c). The genotypes that suffered from copper pre‐treatment under recurring copper excess were not the same genotypes that suffered from copper pre‐treatment under aphid herbivory, since the pre‐treatment ratios on surface area growth rates did not correlate among the environments (Supporting Information: Figure S2.3). These data show that depending on the genotype, copper pre‐treatment can either benefit or harm plant fitness when a stress recurs, with no evidence for transgenerational cross‐resistance under mismatched environments.

We next assessed whether copper excess alleviated stress‐induced morphological changes by analysing area per frond, biomass per frond and area per biomass, three morphological traits that closely correlated with each other (*R* ^2^ > 0.3; *p* < 3 × 10^−7^, mixed effects model, Supporting Information: Figure S2.4). Similar to the patterns observed for *S. polyrhiza* fitness, copper pre‐treatment had the weakest effects on all three morphological parameters when plants were grown under control conditions (Supporting Information: Figure S2.5). Under copper excess and aphid herbivory, copper pre‐treatment either alleviated or increased the stress‐induced morphological changes, depending on the genotype (Supporting Information: Figure S2.5). Taken together, these data show that copper pre‐treatment alters *S. polyrhiza* morphology in a genotype and environment dependent manner, with particularly strong effects of the pre‐treatment under recurring stress.

To assess whether the pre‐treatment induced alterations in *S. polyrhiza* morphology are predictive for plant fitness, we correlated pre‐treatment ratios of the different morphological parameters to the pre‐treatment ratios of *S. polyrhiza* fitness. Under copper excess and aphid herbivory, the stronger the stress‐induced morphological changes were, the more the plant growth rates suffered from copper pre‐treatment (*R* ^2^ > 0.35, *p* > 1 × 10^−4^, mixed effects model, Supporting Information: Figure S2.6). These patterns were absent or even reversed under control conditions (Supporting Information: Figure S2.6). Taken together, these data show that plants that show stronger stress‐induced morphological changes under copper excess and aphid herbivory also suffer more from the copper pre‐treatment in these environments.

### 3.3 Copper Pre‐Treatment Benefits Aphid Fitness

**Figure 3:**
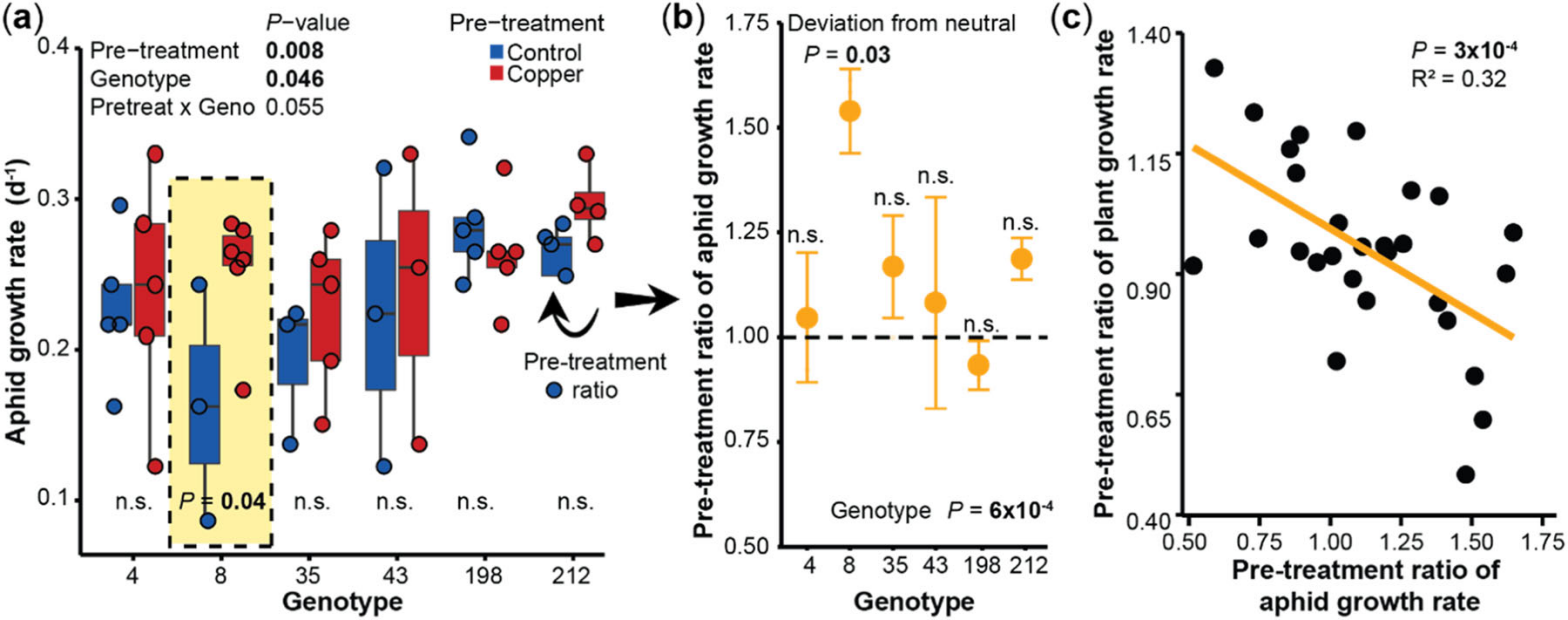
Copper pre‐treatment enhances the fitness of the aphid *Rhopalosiphum nymphaeae*, thereby reducing plant fitness. (a) *R. nymphaeae* reproduced faster on copper than control pre‐treated plants. *p*‐values refer to mixed effects models. Circles display individual data points (b) The pre‐treatment ratios of *R. nymphaeae* growth rates (growth rates on copper pre‐treated plants relative to the mean growth rate on control pre‐treated plants) varied among genotypes. Horizontal dotted line indicates neutral effect of the pre‐treatment. Differences among genotypes refer to mixed effects models. Significant deviations from neutral were analysed with Wilcoxon Rank‐Sum tests. Circles represent the mean value and error bars the standard error. *N* = 3–6. (c) The pre‐treatment ratios of *R. nymphaeae* fitness negatively correlated with the pre‐treatment ratios of plant surface area growth rates. The circles display the replicates of all six genotypes. *p*‐value refers to a mixed effects model. *N* = 28. Geno, genotype; Pretreat, pre‐treatment. [Color figure can be viewed at wileyonlinelibrary.com]

We next assessed whether copper pre‐treatment not only altered *S. polyrhiza* but also *R. nymphaeae* fitness in six different *S. polyrhiza* genotypes (Supporting Information: Table S1). Surprisingly, copper pre‐treatment increased *R. nymphaeae* growth rates in average by 9% (*p* [pre‐treatment] = 0.008, mixed effects model, Figure 3a, Supporting Information: Figure S3.1). These effects were genotype‐specific (*p* [pre‐treatment × genotype] = 0.055, mixed effects model, Figure 3a): while copper pre‐treatment had no effects on *R. nymphaeae* fitness of most plant genotypes, one genotype, SP008, supported more than 50% higher aphid growth rates when plants were pre‐treated with copper (deviation from neutral in genotype SP008: *p* = 0.03, Wilcoxon rank sum test; variation among genotypes: *p* = 6 × 10^−4^, Figure 3b).

To assess whether the increased growth of *R. nymphaeae* due to the copper pre‐treatment reduces *S. polyrhiza* fitness, we correlated the pre‐treatment ratio of aphid fitness with the pre‐treatment ratios of plant surface area growth rate and morphology. The better the aphids grew, the worse the plants performed, and stronger the stress‐induced morphological changes became (*S. polyrhiza* growth rates: *R* ^2^ = 0.32, *p* = 3 × 10^−4^, mixed effects model, Figure 3c; *S. polyrhiza* morphologies: *R* ^2^ > 0.24, *p* < 0.003, mixed effects model, Supporting Information: Figure S3.2). These data show that copper pre‐treatment benefitted aphid fitness on some but not all genotypes, and that the increase in aphid growth reduced plant fitness.

### 3.4 Copper Pre‐Treatment Enhances the Accumulation of Anthocyanins Under Control Conditions and of Jasmonates Under Stress

**Figure 4:**
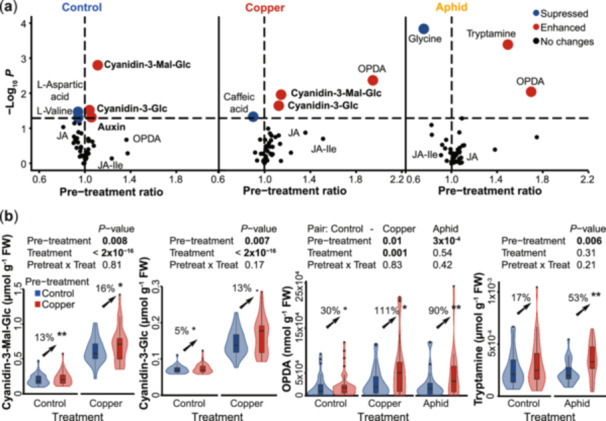
Copper pre‐treatment in *Spirodela polyrhiza* transgenerationally enhances the accumulation of anthocyanins and jasmonate precursors. (a) Copper pre‐treatment transgenerationally elevated the levels of anthocyanins, auxin, tryptamine and the jasmonate precursor 12‐oxo‐phytodienoic acid (OPDA) under copper excess and aphid herbivory. *p*‐values (*y*‐axis) refer to ANOVA test on mixed effects models. Each circle represents the mean value from the replicates of six genotypes per environment. (b) The transgenerationally plastic metabolites were transgenerationally retained rather than primed. Transgenerationally retained refers to significant effects of the pre‐treatment, whereas transgenerationally primed refers to significant interactions of the treatment and pre‐treatment. The data display the replicates from all six genotypes. *p*‐values refer to an ANOVA test on mixed effects models. Asterisks next to the percentages refer to *p*‐values from mixed effects models. < 0.06; * < 0.05; ** < 0.01. *N* = 23–33. Cyanidin‐3‐Glc, cyanidin‐3‐glucoside; Cyanidin‐3‐Mal‐Glc, cyanidin‐3‐malonylglucoside; FW, fresh weight; JA, jasmonic acid; JA‐Ile, jasmonic acid‐isoleucine; OPDA, 12‐oxo‐phytodienoic acid. [Color figure can be viewed at wileyonlinelibrary.com]

To infer which copper‐induced transgenerationally plastic metabolites altered the fitness of *S. polyrhiza* and *R. nymphaeae*, we assessed whether copper pre‐treatment affected the accumulation of different phytohormones, free amino acids and their amines, as well as specialized metabolites derived from the phenylpropanoid pathway using the plant material from the fitness assay of six different genotypes (Supporting Information: Table S1). We first assessed whether copper pre‐treatment altered the accumulation of metabolites under control conditions. Copper pre‐treated plants accumulated 13% and 5% higher levels of the anthocyanins cyanidin‐3‐malonylglucoside and cyanidin‐3‐glucoside, respectively, 7% higher levels of auxin, and 6% lower levels of the free amino acids L‐aspartic acid and L‐valine compared to control pre‐treated plants (*p* < 0.05, mixed effects models, Figure 4).

We next assessed whether copper pre‐treatment enhances the accumulation of metabolites under stress. Under copper excess, copper pre‐treated plants accumulated 16% and 13% higher levels of the anthocyanins cyanidin‐3‐malonylglucoside and cyanidin‐3‐glucoside, respectively, and 111% higher levels of 12‐oxo‐phytodienoic acid (OPDA) than control pre‐treated plants (*p* < 0.05, mixed effects models, Figure 4). The levels of OPDA were also elevated by the copper pre‐treatment under aphid herbivory, where it enhanced OPDA levels in average by 90% (*p* < 0.05, mixed effects model, Figure 4). The strongest increase in OPDA levels upon copper pre‐treatment under aphid herbivory were found in genotype SP008 (Supporting Information: Figure S4.1), the genotype in which the copper pre‐treatment increased aphid growth the most. The OPDA‐derived defence hormones jasmonic acid and jasmonic acid‐isoleucine were not affected by the pre‐treatment in any environment (Figure 4a, Supporting Information: Figure S4.1). Under aphid herbivory, copper pre‐treated plants also accumulated 53% higher levels of the putative defence metabolite tryptamine compared to control pre‐treated plants (*p* < 0.05, mixed effects model, Figure 4). Most of these pre‐treatment‐induced changes in metabolite levels observed under aphid herbivory and copper excess were also seen under control conditions, but to a lower extent: indeed, transgenerational priming (significant interaction of the treatment and pre‐treatment) was not present in any of these metabolites (Figure 4b). To further assess whether these metabolites are primarily transgenerationally retained or primed, we analysed the concentration of these metabolites individually considering genotype as a fixed rather than random effect. This analysis showed that while genotype‐specific transgenerational priming contributes to the elevated levels of these metabolite upon copper pre‐treatment, these elevated levels are transgenerationally retained rather than primed (Supporting Information: Figure S4.2).

We next assessed whether these metabolites were induced by first‐time exposure to copper excess and whether the metabolites were regulated by the copper pre‐treatment in the same direction as under the respective stress. The two cyanidins, whose levels were elevated by about 10% by the copper pre‐treatment under both control conditions and copper excess, were induced by copper excess by approximately 150% (*p* < 2 × 10^−16^, Supporting Information: Figure S4.3). OPDA, whose level was doubled by copper pre‐treatment under copper excess and aphid herbivory, was induced by 50% upon copper excess but not upon aphid herbivory (*p* = 0.002, Supporting Information: Figure S4.3). Tryptamine, whose level was elevated by 50% by the copper pre‐treatment under aphid herbivory, was induced by copper excess by almost 3000%, but was not affected by aphid herbivory (*p* < 2 × 10^−16^, Supporting Information: Figure S4.3). Together, these data show that all transgenerationally plastic metabolites were plastic upon first‐time exposure to copper excess, and that copper exposure mostly reinforces the induction of these metabolites when another stress recurs.

### 3.5 Anthocyanins Likely Protect Plants Against Copper Excess

**Figure 5:**
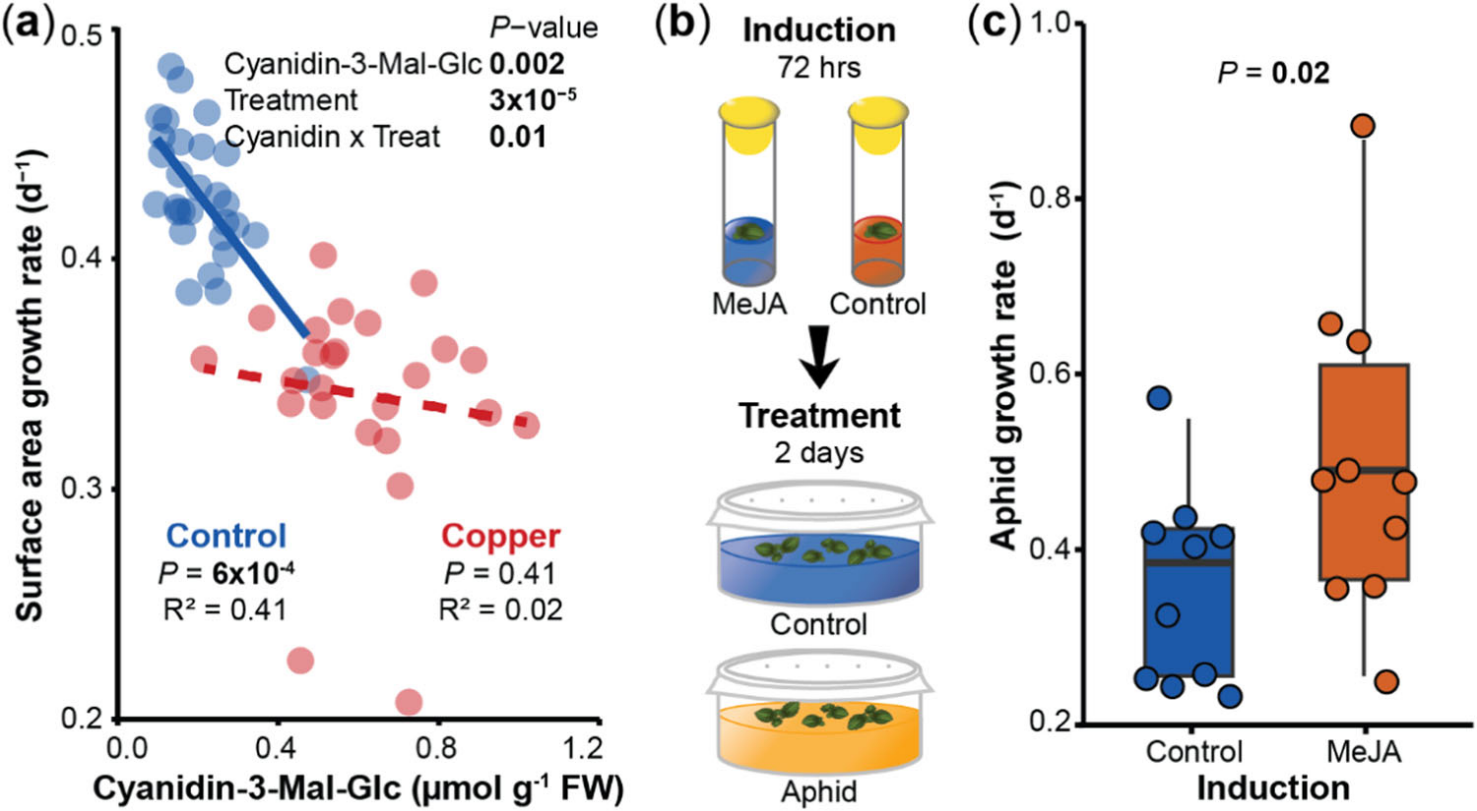
Transgenerationally plastic metabolites benefit *Spirodela polyrhiza* fitness under copper excess but improve *Rhopalosiphum nymphaeae* growth. (a) The levels of cyanidin‐3‐malonylglucoside correlated negatively with plant fitness under control conditions but not under copper excess. Circles display the values of all replicates from the six genotypes from the transgenerational experiment indoors upon 8 days of growth under control conditions or copper excess. *p*‐values refer to mixed effects models. *N* = 27–32. (b) Overview of the induction experiment to test the effect of jasmonates on *R. nymphaeae* growth rates. (c) *R. nymphaeae* produced more offspring when methyl jasmonate was externally applied on plants of genotype SP050. Circles display values of individual samples upon 2 days of growth. *p*‐value refers to a mixed effects model. *N* = 10. Cyanidin‐3‐Mal‐Glc, cyanidin‐3‐malonylglucoside; FW, fresh weight; MeJA, methyl jasmonate; Treat, treatment. [Color figure can be viewed at wileyonlinelibrary.com]

To infer whether the transgenerational plasticity of the specialized metabolites is adaptive, we correlated anthocyanin and tryptamine concentrations with *S. polyrhiza* fitness of the six different genotypes (Supporting Information: Table S1) under control conditions and under the environment in which the enhanced metabolite was inherited. The concentrations of both cyanidins negatively correlated with *S. polyrhiza* growth rates under control conditions but not under copper excess (cyanidin‐3‐malonyglucoside: *p* = 6 × 10^−4^ in control and *p* = 0.41 in copper, interaction: *p* = 0.01, mixed effects models, Figure 5a; cyanidin‐3‐glucoside: Supporting Information: Figure S5.1). Thus, the production of cyanidins is likely costly under control conditions but these costs are alleviated under copper excess. The concentrations of tryptamine did not correlate with plant fitness under control conditions or aphid herbivory (*p* [tryptamine × treatment] = 0.91, mixed effects model, Supporting Information: Figure S5.1).

### 3.6 External Application of Methyl Jasmonate in Plants Increases OPDA Concentrations and Benefits Aphid Growth Rates

We next tested the effect of jasmonates on *R. nymphaeae* growth rates, as the jasmonate precursor OPDA was elevated by the copper pre‐treatment under aphid herbivory, and as pre‐treatment ratios of OPDA and jasmonates were positively correlated (*R* > 0.14, *p* ≤ 0.05, Supporting Information: Figure S5.2). Thereto, we measured the growth rates of *R. nymphaeae* when *S. polyrhiza* genotype SP050 was pre‐treated for 3 days with either a mock solution or methyl jasmonate (Figure 5b), the latter of which induced the levels of OPDA, jasmonic acid and jasmonic acid‐isoleucine by 640%, 200% and 240%, respectively (Supporting Information: Figure S5.3). Surprisingly, *R. nymphaeae* had 39% higher growth rates within 48 h when plants were pre‐treated with methyl jasmonate (*p* = 0.02, mixed effects model, Figure 5c). To confirm this unexpected finding, we repeated the experiment, yielding similar results (*p* = 0.06, mixed effects model, Supporting Information: Figure S5.4). Taken together, these data show that transgenerational plasticity of anthocyanins is likely adaptive, whereas transgenerational plasticity of jasmonates may be maladaptive.

## 4 Discussion

In this study, we showed that previous exposure of *S. polyrhiza* to copper excess can alter the fitness of both the plant and its native herbivore. On one hand, previous exposure to copper excess benefitted *S. polyrhiza* fitness in some genotypes under recurring stress conditions, likely because copper excess induced defensive anthocyanins, and these elevated levels were transgenerationally retained. On the other hand, previous exposure to copper also benefitted the fitness of a native aphid herbivore and thereby harmed *S. polyrhiza* fitness, a phenomenon that was likely mediated by transgenerationally elevated levels of OPDA, a jasmonate precursor. Thus, these data reveal that pollutants that elicit transgenerational plasticity in plants can have unexpected consequences on both the fitness of plants and their herbivores.

Inheritance of traits across generations is an increasingly recognized phenomenon in plants. While trait inheritance is common across one unexposed generation (maternal effects), and can persist across two generations (Macartney et al. 2023; Yin et al. 2019), there is little evidence that traits are transgenerationally inherited (Anastasiadi et al. 2021), meaning being plastic for three or more generations (Groot et al. 2017; Huber, Gablenz, and Höfer 2021; Lin et al. 2024; Van Antro et al. 2023). Here, we demonstrate such transgenerational plasticity. First, copper pre‐treatment altered plant morphologies in an environment‐ and genotype dependent manner. Second, copper excess induced the accumulation of anthocyanins, and these elevated levels were transgenerationally retained. Third, copper excess elevated the levels of OPDA, and these elevated levels were retained under recurring copper excess and aphid herbivory. Together, these data show that in *S. polyrhiza*, morphological and metabolic traits can be plastic for more than three generations.

We did not attempt to identify the molecular mechanisms underlying trait inheritance. It is very likely that the transgenerational variation was due to plasticity rather than underlying genetic variation, because *S. polyrhiza* reproduces clonally and accumulates only one point mutation every 80 generations (Xu et al. 2019). Plasticity that persists for three or more generations is thought to rely on the inheritance of epigenetic marks or microbes rather than the transmission of substances (Jablonka and Raz 2009; Perez and Lehner 2019). To reduce the effect of microbes, we performed our indoor experiments under axenic conditions. Under axenic conditions, copper pre‐treatment no longer protected genotype SP004 under recurring stress as observed outdoors, possibly because of vertical transmission of microbes outdoors. In general, the transgenerational effects on fitness were weaker under axenic conditions indoors compared to the ones outdoors, and also weaker than previous transgenerational studies with *S. polyrhiza* under non‐axenic environments indoors (Huber, Gablenz, and Höfer 2021); nevertheless, transgenerational plasticity under axenic conditions indoors was present. Thus, it is possible that epigenetic inheritance mediates the observed transgenerational responses. Indeed, the closely related duckweed *Lemna minor* transmits stress‐induced variation in DNA methylation across multiple generations (Van Antro et al. 2023), similar to other clonally reproducing plants whose methylome is altered by stress across several generations (Latzel, Rendina González, and Rosenthal 2016). Future experiments that investigate transgenerational variation in epigenetic marks, and that genetically manipulate the epigenetic machinery or epigenetically regulated genes are required to elucidate the molecular mechanisms of transgenerational plasticity.

Transgenerational plasticity is hypothesized to benefit plant fitness under recurring stress by either retaining elevated levels of defences or priming of the defences. In our study, we found that copper‐induced levels of anthocyanins were transgenerationally retained rather than transgenerationally primed. The anthocyanins likely protected *S. polyrhiza* under copper excess, because anthocyanin concentrations were negatively correlated with plant fitness under control conditions but not under copper excess. The observed protective function is in line with previous studies showing that anthocyanins are induced under abiotic stresses and increase plant stress tolerance (Ai et al. 2018; Naing and Kim 2021). Taken together, these data show that transgenerational plasticity can be adaptive under matching environments by retaining stress‐induced levels of defensive metabolites.

While our data show that transgenerational plasticity can benefit plant fitness under matched environments, we also obtained evidence that transgenerational plasticity can reduce plant fitness under unmatched environments. First, pre‐treatment ratios of *S. polyrhiza* growth rates did not correlate across the tested environments, suggesting that transgenerational cross‐resistance is not common. This is in line with previous multi‐generational studies, in which infection of the pathogens *P. syringae* and *Plectosphaerella cucumerina* increased the disease resistance in *A. thaliana* offspring in matched environments, but increased susceptibility in unmatched environments (López Sánchez et al. 2021). Together with our data, these patterns suggest that transgenerational plasticity may involve trade‐offs, by increasing fitness in one environment but decreasing fitness in another environment. Such trade‐offs could also explain why in our study transgenerational plasticity varied among genotypes.

Second, we found that copper pre‐treatment benefitted *R. nymphaeae* fitness—in some genotypes up to 50%—and increased aphid growth was associated with reduced *S. polyrhiza* fitness. Aphids likely grew better because OPDA, a jasmonate precursor, was induced by copper excess and these elevated levels were transgenerationally retained. Although boosting of the jasmonate pathway is hypothesized to reduce aphid growth (Aslam et al. 2022; Züst and Agrawal 2016), *R. nymphaeae* grew 40% better when *S. polyrhiza* was pre‐treated with methyl jasmonate. Although we used different genotypes for the external methyl jasmonate application and the transgenerational experiments, it is likely that methyl jasmonate has similar effects on plant resistance and aphid growth across genotypes, as the jasmonate pathway is conserved (Katsir et al. 2008). Similarly, in *Sorghum bicolor*, external application of jasmonates promoted aphid feeding and colonization, and plants with impaired jasmonate biosynthesis supported lower aphid population growth (Grover et al. 2022). In our study, methyl jasmonate induced not only jasmonic acid and jasmonic acid‐isoleucine but also OPDA, the latter of which can have jasmonate‐independent functions (Jimenez Aleman et al. 2022). Thus, we do not know whether jasmonate‐dependent or ‐independent mechanisms of OPDA accounted the increased aphid growth upon copper pre‐treatment and methyl jasmonate application. Nevertheless, our study highlights that not all aphids are susceptible to jasmonate‐induced defences, and that jasmonates may be a key hormone in orchestrating transgenerational plasticity (Rasmann et al. 2012). Transgenerational plasticity in jasmonates can, however, be maladaptive in unmatched environments, by promoting aphid growth and reducing plant fitness.

The increased aphid growth due to the previous copper exposure could have far‐reaching consequences on species composition and ecosystem functioning: duckweeds can quickly cover water surfaces, causing dark and anoxic conditions (Scheffer et al. 2003). Increased growth of *R. nymphaeae* could counteract these conditions, and thereby alter species composition in these aquatic ecosystems, which in turn, could alter ecological and evolutionary processes on land. Such interactions of aquatic and terrestrial ecosystems may be common: for instance, terrestrial flowering plants received more pollinators and were less pollen limited when the ponds nearby contained fishes that prey on dragon fly larvae, as mature dragon flies consume insect pollinators (Knight et al. 2005). Thus, transgenerational plasticity of an aquatic, clonally reproducing key stone species, such as duckweeds, may have unexpected consequences on species interactions and eco‐evolutionary dynamics both in water and on land.

Taken together, our study shows that a common aquatic pollutant induces defence hormones and metabolites, and that elevated levels of these defences are retained across multiple generations. Transgenerational plastic defences can benefit plant fitness if the same stress recurs but can also harm plant fitness in the presence of herbivores by promoting herbivore population growth. Our results thus provide evidence that a pollutant early in the growing season can boost herbivore growth later in the season, thus revealing unexpected consequences of transgenerational plasticity on species interactions.

## Conflicts of Interest

The authors declare no conflicts of interest.

## Supporting information

Supporting information.

## Data Availability

All raw data and R scripts for the analyses and plots within this study are deposited in https://github.com/Plant-Evolutionary-Ecology-Lab/Transgenerational-Cross-Resistance.
