# Supplementary material for: Copper‐Induced Transgenerational Plasticity in Plant Defence Boosts Aphid Fitness
Source: Plant Cell Environ. 2025 Jan 27;48(6):3997–4010. doi: 10.1111/pce.15406 (PMC12050396; doi:10.1111/pce.15406)
Supplement: Supplementary file 1 — Supporting information. [file PCE-48-3997-s001.pdf]

Copper-induced transgenerational plasticity in plant defence boosts aphid fitness

Alexandra Chávez, Anne Schreyer, Pauline Prüsener, Martin Schäfer, Shuqing Xu, Meret Huber

Supporting Information

Supporting figures 3

Figure S1. Pre-treatment phase in outdoor conditions. 3

Figure S2.1. Propagation diagrams of six different genotypes of *Spirodela polyrhiza* under axenic conditions. 4

Figure S2.2. Surface area growth rates of *Spirodela polyrhiza* varied among the different stress treatments. 5

Figure S2.3. Copper pre-treatment in *Spirodela polyrhiza* does not lead to cross-resistance. 6

Figure S2.4. Morphological traits of *Spirodela polyrhiza* correlate among each other.......... 7

Figure S2.5. Copper excess indoors alters *Spirodela polyrhiza* morphology in a genotype and treatment dependent manner. 8

Figure S2.6. Pre-treatment effects on plant morphology were predictive for plant fitness, particularly under copper excess and aphid herbivory.......... 9

Figure S3.1. Copper pre-treatment on *Spirodela polyrhiza* tends to increase the growth rate of the aphid *Rhopalosiphum nymphaeae*. 11

Figure S3.2. The more *Rhopalosiphum nymphaeae* benefitted from the copper pre-treatment, the stronger the stress-induced morphological changes became in *Spirodela polyrhiza*. 11

Figure S4.1. Copper pre-treatment enhances the concentrations of 12-oxo-phytodienoic acid in *Spirodela polyrhiza*, but not jasmonates under aphid herbivory. 12

Figure S4.2. Copper excess transgenerationally retains copper-induced levels of anthocyanins and jasmonates..........13

Figure S4.3. First time exposure to copper excess enhances the levels of transgenerational plastic metabolites in *Spirodela polyrhiza*. 14

Figure S5.1. Transgenerationally plastic cyanidins have an adaptive role in plants against copper, but not tryptamine against aphid herbivory. 15

Figure S5.2. Pre-treatment ratios of jasmonates and its precursor 12-oxo-phytodienoic acid (OPDA) correlate among each other. 16

Figure S5.3. Exogenous application of methyl jasmonate in *Spirodela polyrhiza* elevates the concentrations of jasmonates and their precursor. 17

Figure S5.4. External application of methyl jasmonate to *Spirodela polyrhiza* tends to increase the growth rate of the aphid *Rhopalosiphum nymphaeae*. 18

Supporting tables..19

Table S1. Genotypes of *Spirodela polyrhiza* used in the experiments. 19

Table S2. MRM-settings and retention times of a putative chlorogenic acid isomer. 19

Table S3. Copper excess treatment effects *Spirodela polyrhiza* surface area growth rate independently of the outdoor pre-treatment. 19

Supporting methods 20

Methods S1. Variables and models used to characterize the effects that the pre-treatment has on *Spirodela polyrhiza* fitness and morphology. 20

Methods S2. Variables and models used to characterize the effect of the pre-treatment on *Spirodela polyrhiza* metabolites. 22

Methods S3. Assessing the effects of *Spirodela polyrhiza* induced jasmonates in the growth rates of *Rhopalosiphum nymphaeae*. 24

Methods S4. Variables to assess the effects of methyl jasmonate on *Rhopalosiphum nymphaeae* growth rates. 24

Equations used for the transformation of data 26

Equation S1. Relative growth rate (Hunt, 1982). 26

Equation S2. Pre-treatment ratio (Huber et al., 2021). 26

Supporting figures

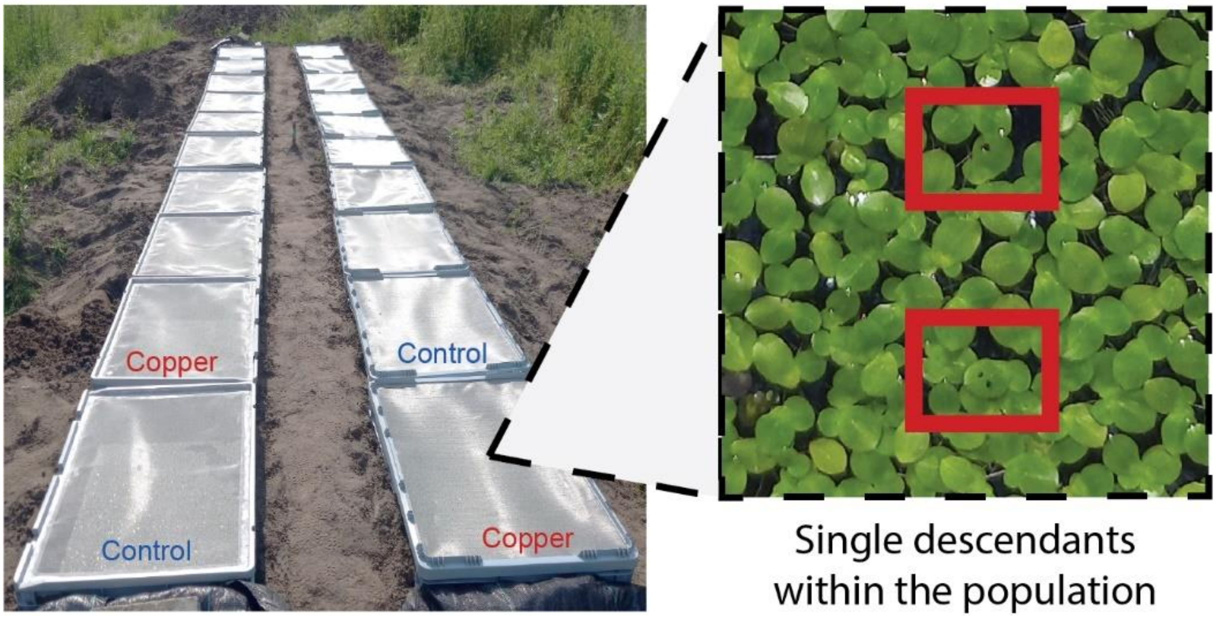

Figure S1. Pre-treatment phase in outdoor conditions. Outdoors, *S. polyrhiza* genotype SP004 was grown under copper and control conditions as part of the pre-treatment phase. Within the plant population single descendant lineages were marked and followed for five generations. Marking was done as: first generations, one dot; second generation, two dots; third generation, three dots; fourth generation one dot; fifth generation two dots; sixth generation starting fitness assays, three dots. Once the new generation naturally detached from the previous generation, the older generation was taken out of the experiment. N = 10.

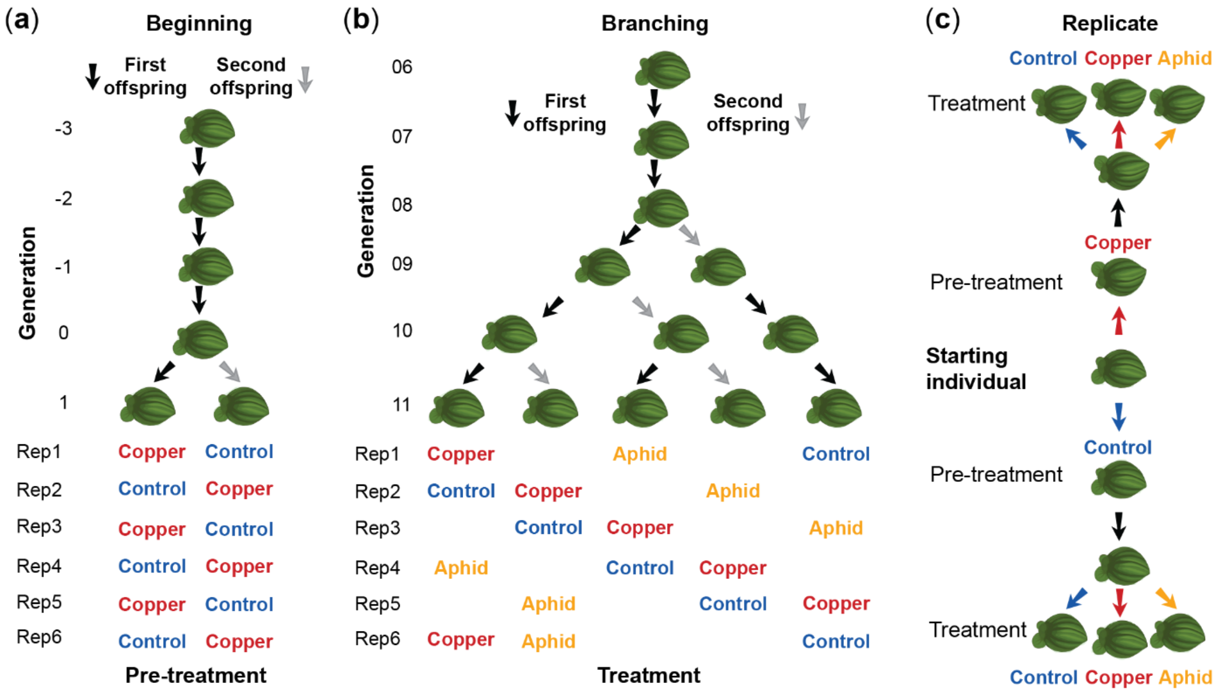

Figure S2.1. Propagation diagrams of six different genotypes of *Spirodela polyrhiza* under axenic conditions. (**a**) The first and second daughters of single descendants were placed into copper and control condition to start the pre-treatment. (**b**) Single descendants indoors were branched after copper pre-treatment using the first and the second offspring and placed into determined treatments depending on the replicate number. (**c**) Each of the six starting individuals were branched into the different pre-treatment and treatment environments. Rep = Replicate.

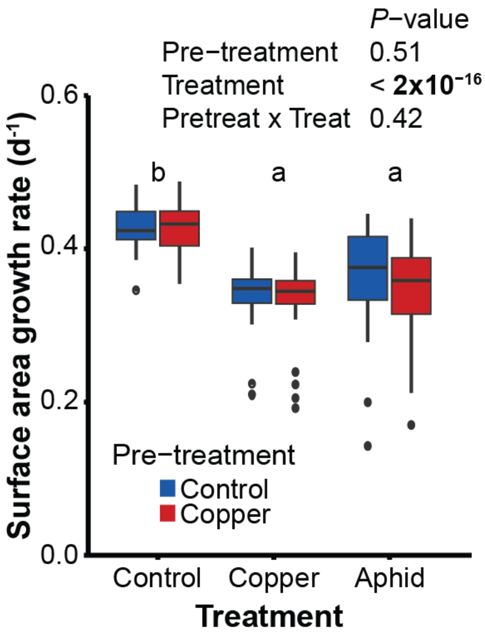

Figure S2.2. Surface area growth rates of *Spirodela polyrhiza* varied among the different stress treatments. The replicates from all six different *S. polyrhiza* genotypes showed fitness differences due to the treatment, but not the pre-treatment environments. *P*-values refer to an ANOVA test on a mixed effects model. Pairwise comparisons among treatments come from least-squares means on treatments of the mixed effects model. N = 24-32.

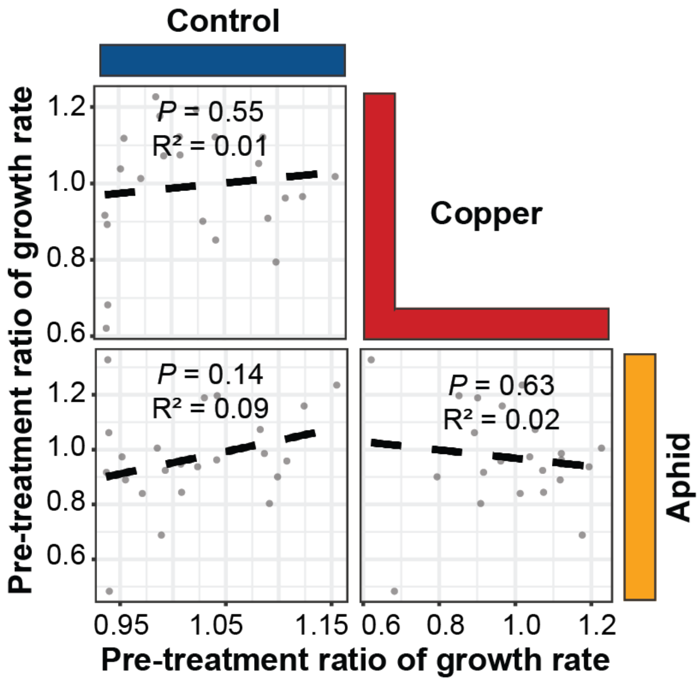

Figure S2.3. Copper pre-treatment in *Spirodela polyrhiza* does not lead to cross-resistance. The pre-treatment ratios on surface area growth rates (growth rates of copper pre-treated plants relative to the mean growth rate of control pre-treated plants per treatment) did not correlate among treatments when considering the replicates of all six genotypes. Dots represent individual values. *P*-values refer to mixed effects models. N = 23.

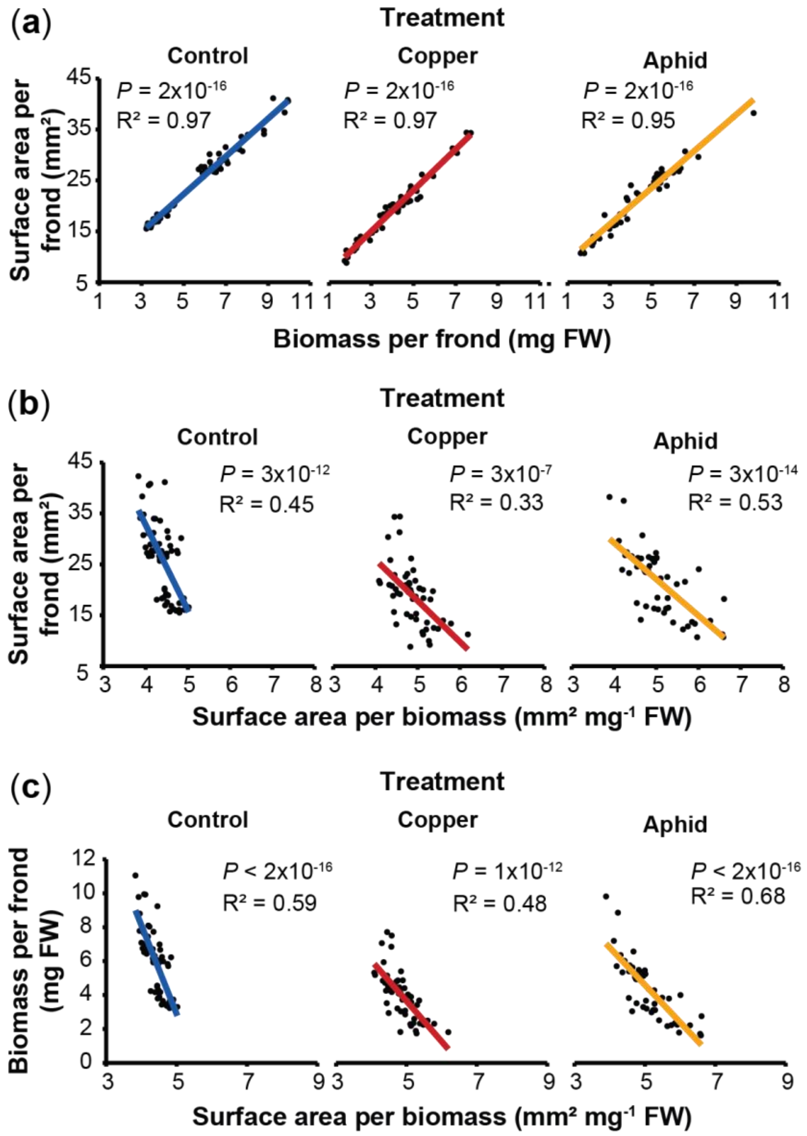

Figure S2.4. Morphological traits of *Spirodela polyrhiza* correlate among each other. (**a**) Biomass per frond positively correlated with surface area per frond. (**b**) Surface area per biomass negatively correlated with surface area per frond in all environments. (**c**) Surface area per biomass negatively correlated with biomass per frond in all environments. Dots represent individual replicates of all six genotypes. Correlation lines are only shown for significant correlations. *P*-values on right of the panels refer to ANOVA tests on mixed effects models. N = 52-60. Morph = morphology, Treat = treatment, FW = fresh weight.

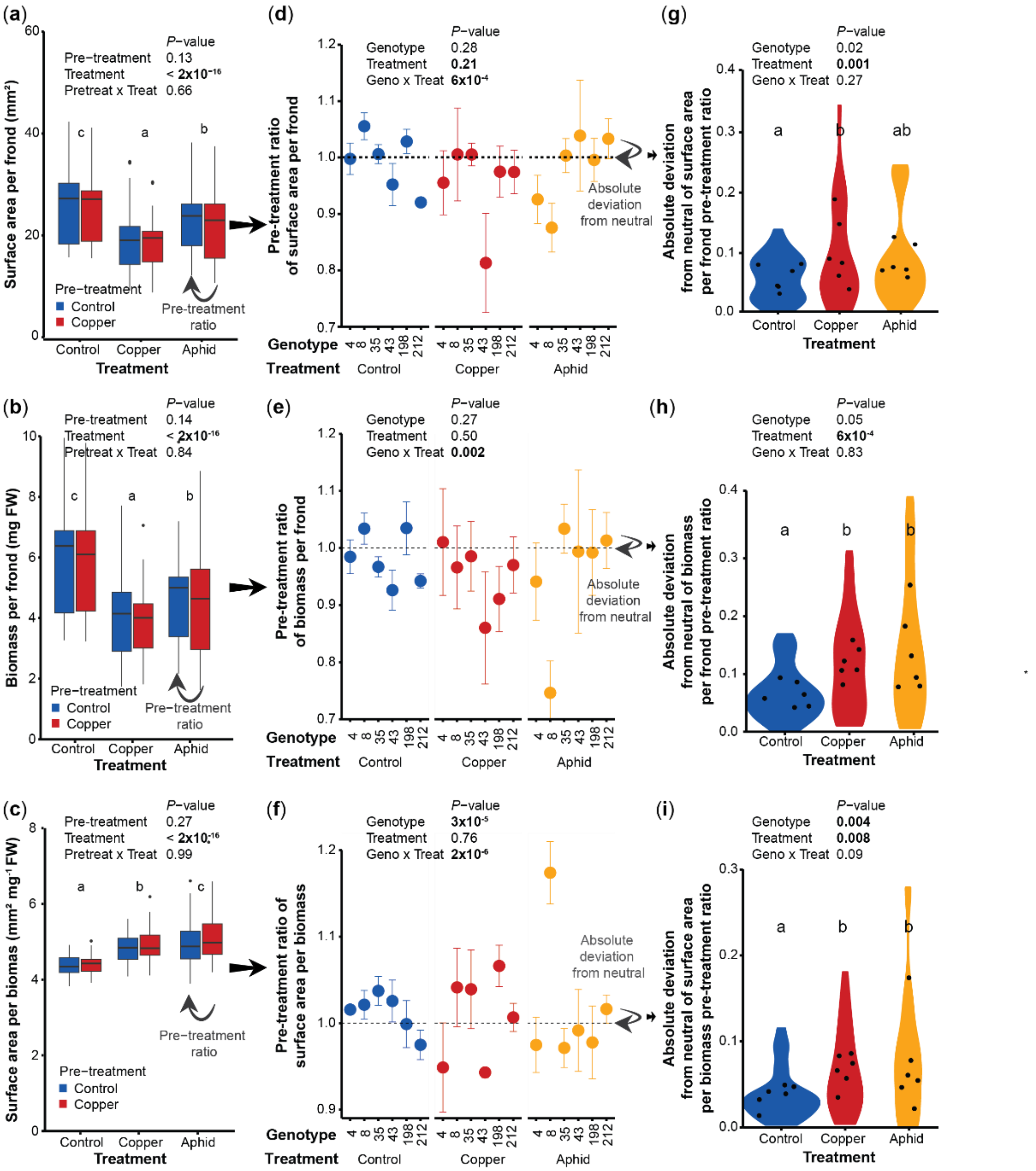

Figure S2.5. Copper excess indoors alters *Spirodela polyrhiza* morphology in a genotype and treatment dependent manner. (**a**) Surface area per frond, (**b**) biomass per frond and (**c**) surface area per biomass varied among treatments when considering the replicates of all six genotypes. Pairwise comparisons among treatments refer to least-squares means. *P*-values refer to ANOVA tests on mixed effects models. N = 24-32. (**d**) Surface area per frond, (**e**) biomass per frond and (**f**) surface area per biomass pre-treatment ratios (phenotype of copper pre-treated plants relative to the mean phenotype of control pre-treated plants per treatment) depended on the genotype and treatment, and their interaction. Dots represent the mean value and error bars the standard error per genotype and

treatment. *P*-values refer to ANOVA tests on mixed effects models. N = 4-6. (**g**) Surface area per frond, (**h**) biomass per frond and (**i**) surface area per biomass pre-treatment ratios of replicates from all six genotypes deviated more from neutral under copper excess and aphid herbivory than under control conditions. N = 24-32. *P*-values refer to ANOVA tests on mixed effects models. Pretreat = Pre-treatment, Treat = Treatment, Geno = Genotype.

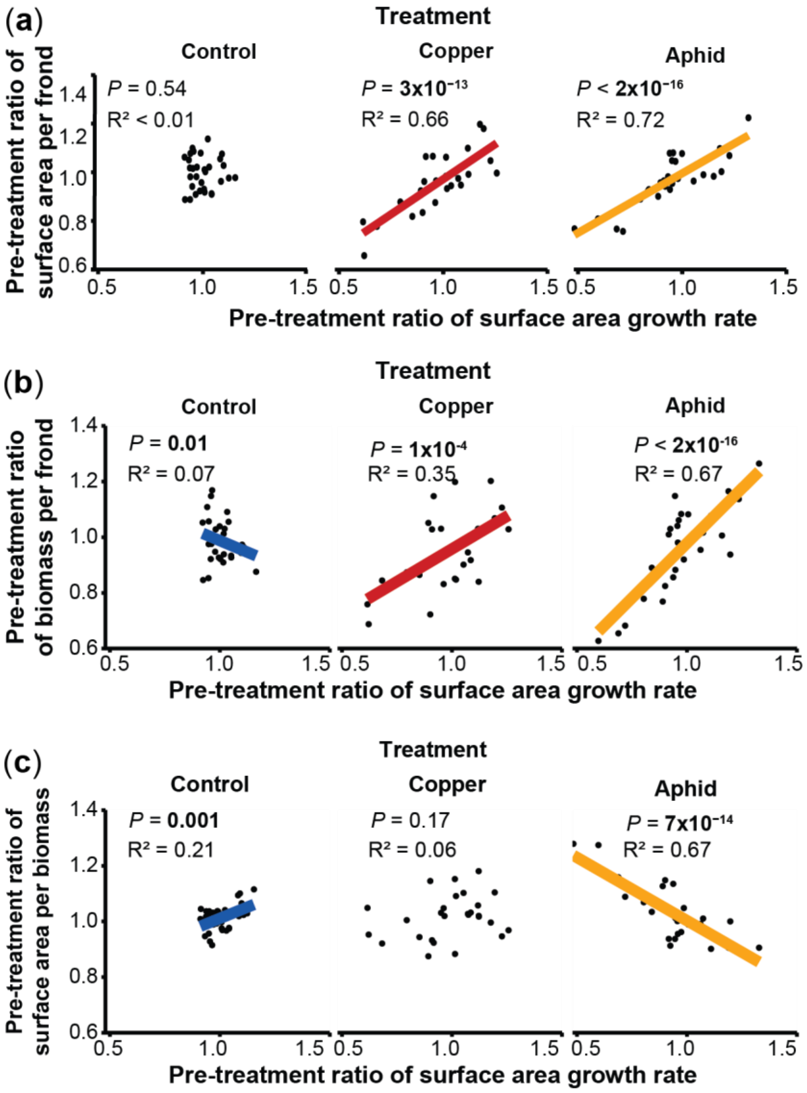

Figure S2.6. Pre-treatment effects on plant morphology were predictive for plant fitness, particularly under copper excess and aphid herbivory. Pre-treatment ratios on surface area growth rates correlated to the pre-treatment ratios on (**a**) surface area per frond, (**b**) biomass per frond, and (**c**) surface area per biomass. Dots represent individual replicates of all six genotypes. Correlation lines are only shown for significant correlations. *P*-values

within a treatment refer to ANOVA tests on mixed effects models. mixed effects models. N = 27-32.

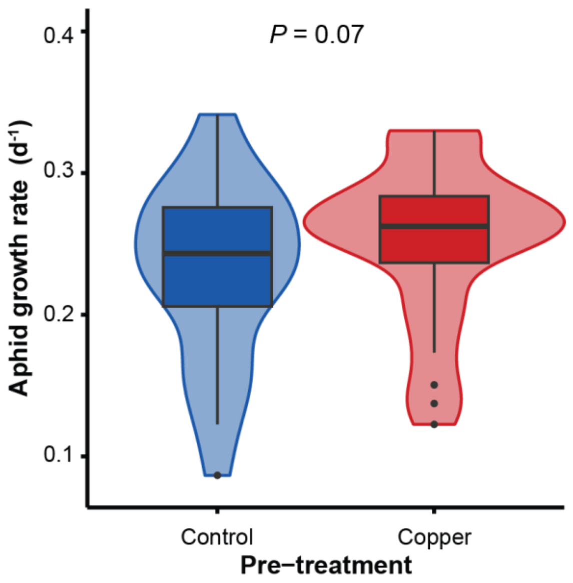

Figure S3.1. Copper pre-treatment on *Spirodela polyrhiza* tends to increase the growth rate of the aphid *Rhopalosiphum nymphaeae*. Copper pre-treatment on *S. polyrhiza* increased aphid growth on the replicates of all six genotypes. *P*-value refers to an ANOVA performed on a mixed effects model. N = 24-28.

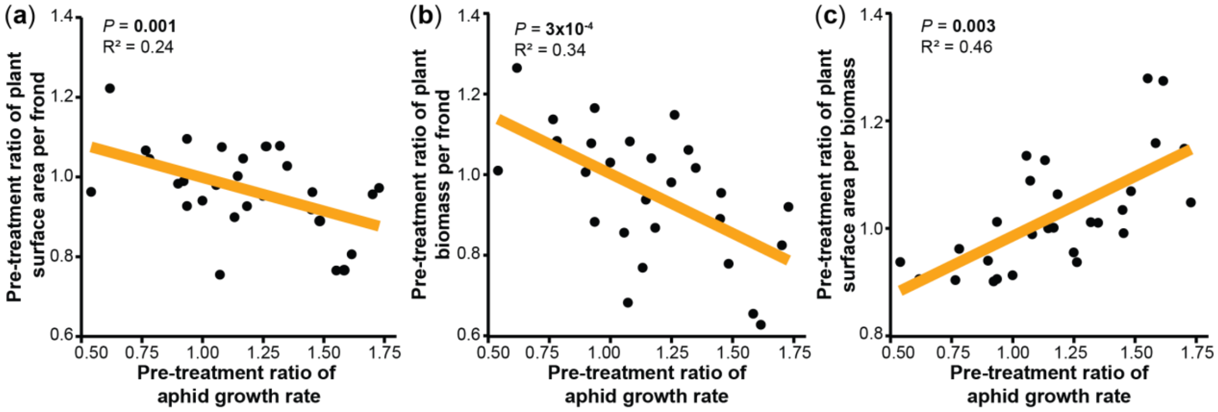

Figure S3.2. The more *Rhopalosiphum nymphaeae* benefitted from the copper pre-treatment, the stronger the stress-induced morphological changes became in *Spirodela polyrhiza*. Correlation between the pre-treatment ratios of *R. nymphaeae* growth rates (growth rates on copper pre-treated plants relative to the mean growth rate on control pre-treated plants) and the pre-treatment ratios of (**a**) surface area per frond, (**b**) biomass per frond, and (**c**) surface area per biomass. Dots represent individual replicates of all six genotypes. *P*-values refers to ANOVA tests performed on mixed effects models. N = 28.

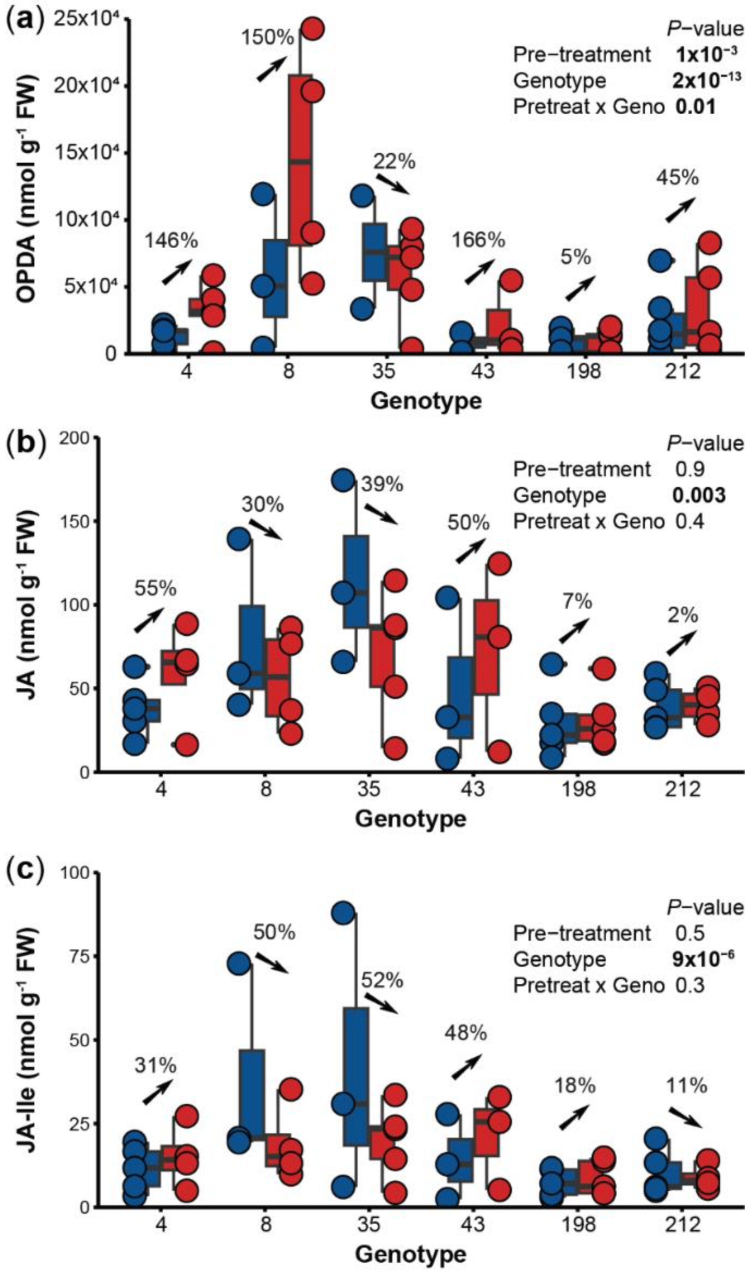

Figure S4.1. Copper pre-treatment enhances the concentrations of 12-oxo-phytodienoic acid in *Spirodela polyrhiza*, but not jasmonates under aphid herbivory. (**a**) Under aphid herbivory, **c**opper pre-treatment enhanced 12-oxo-phytodienoic acid in a genotype dependant manner, with the highest concentrations observed in SP8, genotype that carried the greatest aphid growth rates. (**b**) Copper pre-treatment did not affect jasmonic acid concentrations, nor (**c**) jasmonic acid-isoleucine concentrations. Dots represent individual replicates per genotype. *P*-values refer to ANOVA on mixed effects models. N = 23-32. OPDA = 12-oxo-phytodienoic acid, JA = jasmonic acid, JA-Ile = jasmonic acid-isoleucine, FW = fresh weight.

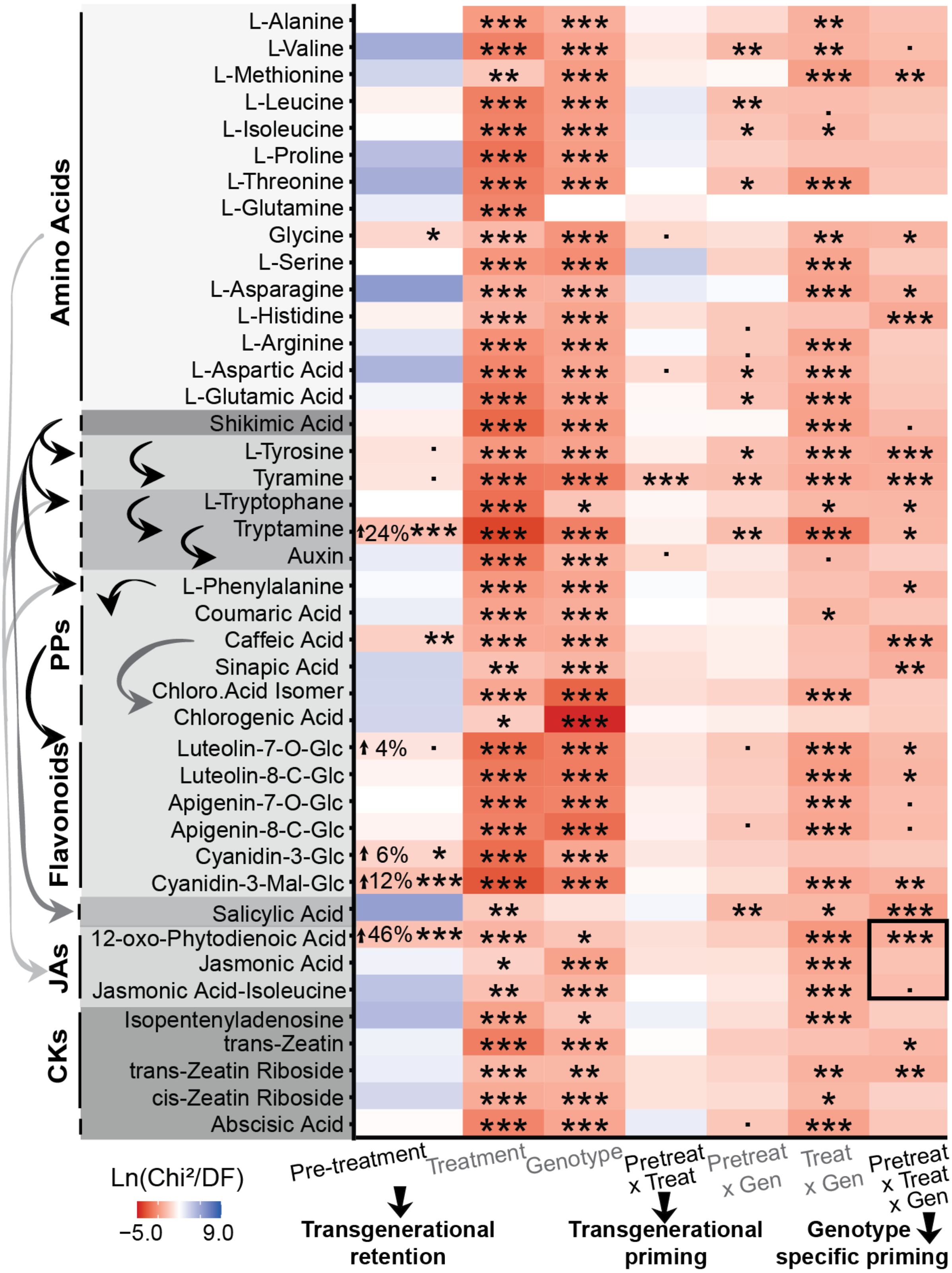

Figure S4.2. Copper excess transgenerationally retains copper-induced levels of anthocyanins and jasmonates. Heatmap displays the Chi^2 values relative to their degrees of freedom (DF) obtained from ANOVA tests performed on mixed effects models of the pre-treatment, treatment, genotype effects on metabolite concentrations. Numbers within the heat map refer to the fit mean metabolite levels between the copper to control pre-treated

plant. Upward pointing arrow refers to increased levels upon copper pre-treatment. *P*-values refer to ANOVA tests on mixed effects models. The different shades of grey cluster metabolites that have a shared biosynthetic pathway, and arrows connect precursors and derivates within and between the assessed pathways. Grey arrows connect indirect precursors and derivates. *P*-values significance: **-** < 0.1, * < 0.05. ** < 0.01, *** < 0.001. N = 3-6 per each of the six genotypes. Cyanidin-3-Glc = cyanidin-3-glucoside, Cyanidin-3-Mal-Glc = cyanidin-3-malonyglucoside, Glc = glucoside, PPs = phenylpropanoids, JAs = jasmonates, CKs = cytokines.

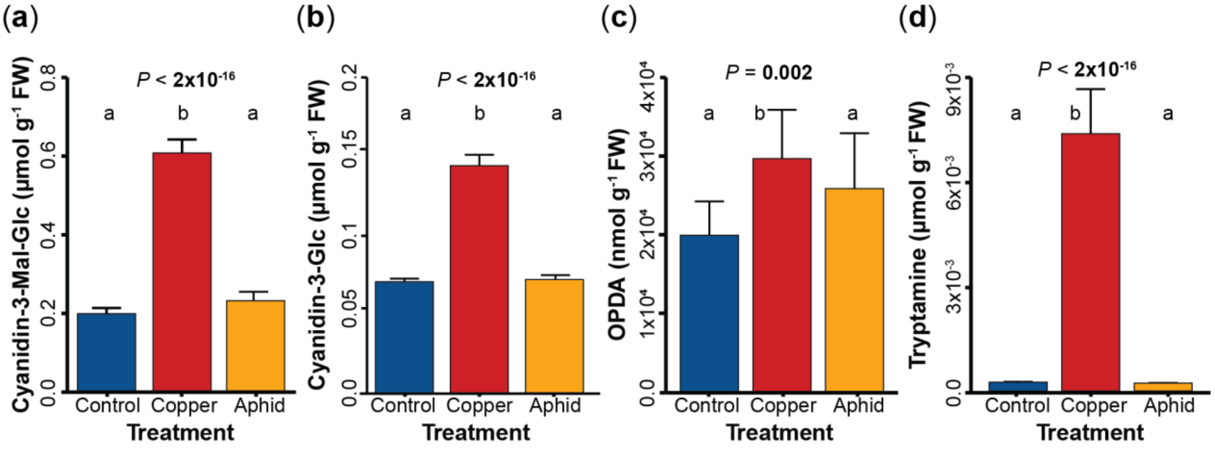

Figure S4.3. First time exposure to copper excess enhances the levels of transgenerational plastic metabolites in *Spirodela polyrhiza*. Copper excess, but not aphid herbivory elevated the levels of (**a**) cyanidin-3-malonylglucoside, (**b**) cyanidin-3-glucoside, (**c**) 12-oxo-phytodienoic acid and (**d**) tryptamine. *P*-values refer to ANOVA on mixed effects models. Pairwise comparisons were obtained with least-squares means. The data display the mean and standard errors of the replicates of all six genotypes. N = 23-32. Cyanidin-3-Mal-Glc = cyanidin-3-malonylglucoside, cyanidin-3-Glc = cyanidin-3-glucoside, OPDA = 12-oxo-phytodienoic acid, FW = fresh weight.

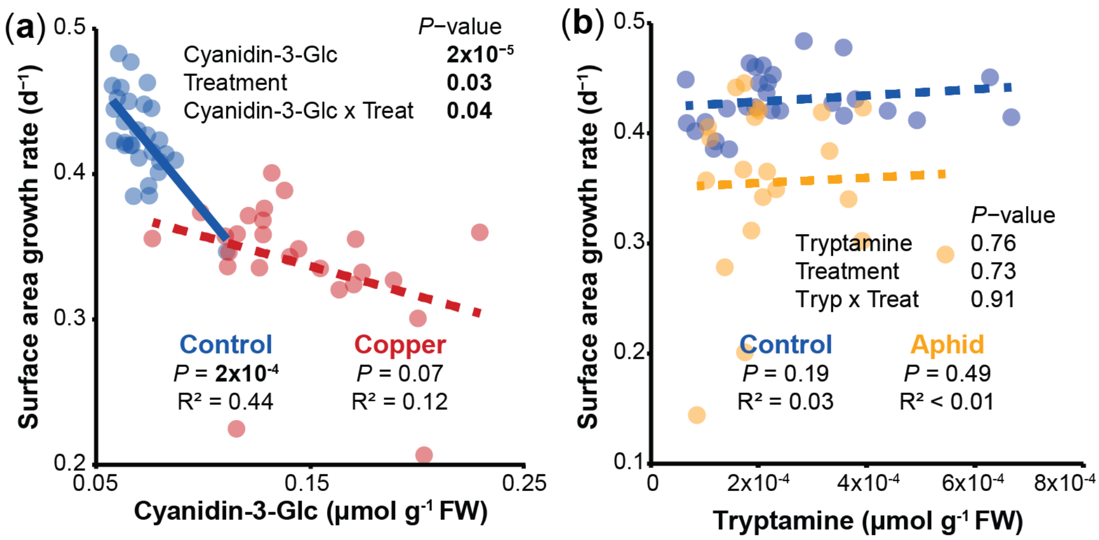

Figure S5.1. Transgenerationally plastic cyanidins have an adaptive role in plants against copper, but not tryptamine against aphid herbivory. (**a**) The levels of cyanidin-3-glucoside negatively correlated with plant fitness under control but not copper conditions. (**b**) Tryptamine levels did not correlate with plant fitness neither under control conditions nor herbivory of the aphid *R. nymphaeae*. Circles display individual replicates of all six genotypes. *P*-values refer to ANOVA tests on mixed effects models. N = 27-32. Cyanidin-3-Glc and Cya3Glc = cyanidin-3-glucoside, Tryp = Tryptamine, Treat = Treatment.

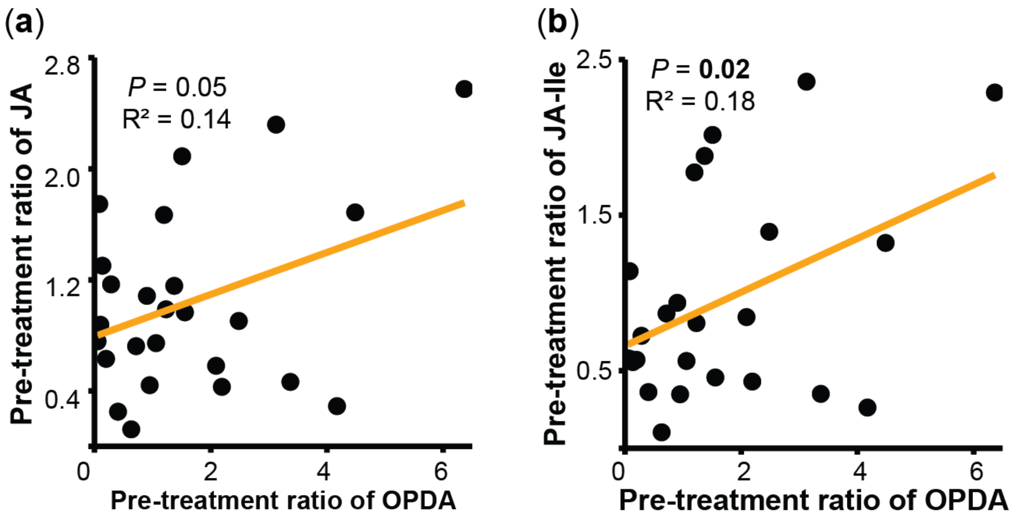

Figure S5.2. Pre-treatment ratios of jasmonates and its precursor 12-oxo-phytodienoic acid (OPDA) correlate among each other. Pre-treatment ratios (metabolite concentrations of copper pre-treated plants relative to the mean metabolite concentration of control pre-treated plants) of OPDA positively correlated with the pre-treatment ratios of (**a**) jasmonic acid and (**b**) jasmonic acid isoleucine under aphid herbivory treatment. Circles display individual replicates of all six genotypes. *P*-values refer to ANOVA tests on mixed effects models. N = 25. JA = jasmonic acid, JA-Ile = jasmonic acid isoleucine, OPDA =12-oxo-phytodienoic acid.

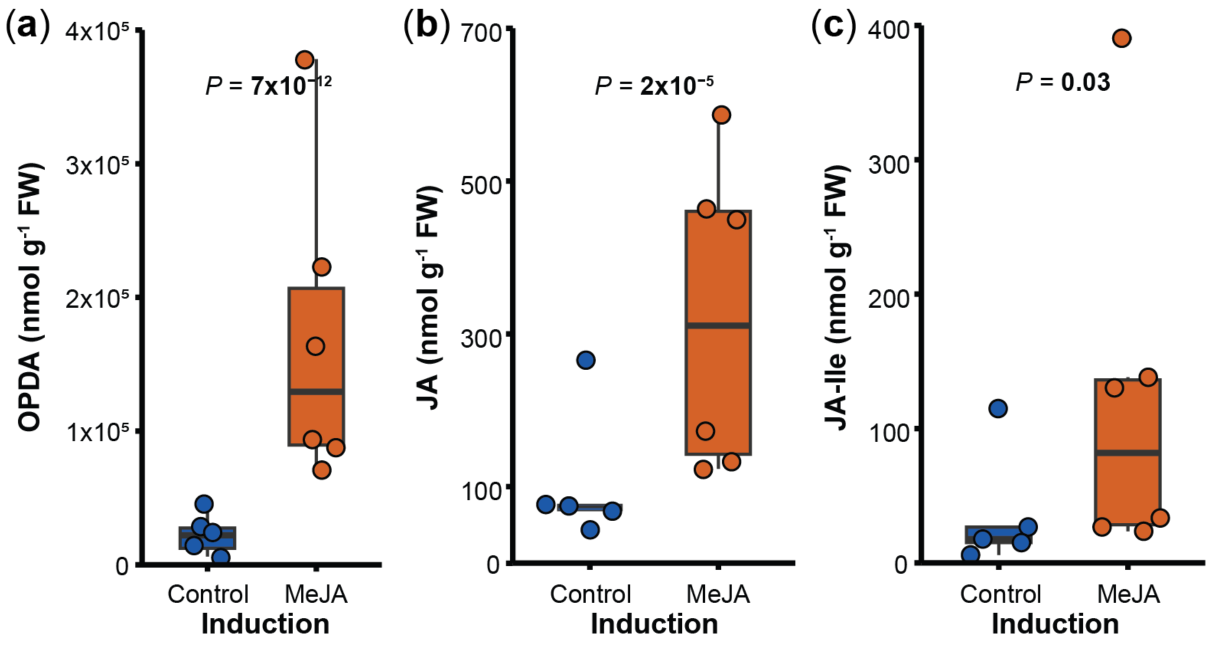

Figure S5.3. Exogenous application of methyl jasmonate in *Spirodela polyrhiza* elevates the concentrations of jasmonates and their precursor. Application of methyl jasmonate on *S. polyrhiza* genotype SP050 enhanced the concentrations of (a) 12-oxo-phytodienoic acid, (b) jasmonic acid and (c) jasmonic acid-isoleucine. *P*-values refer to ANOVA tests on mixed effects models. Circles display individual data points. N = 5-6. JA = jasmonic acid, JA-Ile = jasmonic acid isoleucine, OPDA =12-oxo-phytodienoic acid.

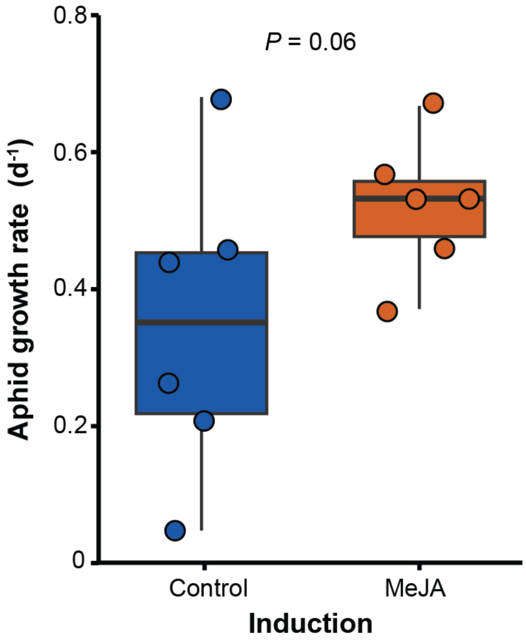

Figure S5.4. External application of methyl jasmonate to *Spirodela polyrhiza* tends to increase the growth rate of the aphid *Rhopalosiphum nymphaeae*. Repeating the application of methyl jasmonate on *S. polyrhiza* genotype SP050 tended to favour aphid growth after two days of assays. *P*-value refers to an ANOVA test on a mixed effects model. Circles display individual data points. N= 6. MeJA = methyl jasmonate, 12-oxo-phytodienoic acid = OPDA.

Supporting tables

Table S1. Genotypes of *Spirodela polyrhiza* used in the experiments.

Clone ID	Accession ID	Genetic cluster	Continent	Country	Latitude	Longitude	NCBI accession ID	Experiment
7498	SP004	America	North America	USA	35.994033	-78.898619	SAMN09429369	Outdoor/Indoor
8683	SP008	America	Africa	Kenya	0.0880828	38.189978	SAMN09429399	Indoor
0109	SP035	SE Asia	Asia	China	30.196789	103.5065	SAMN09429365	Indoor
9497	SP043	India	Asia	India	9.9252007	78.119775	SAMN09429373	Indoor
0090	SP050	SE Asia	Asia	China	30.410755	104.54677	SAMN09429379	Induction
NA	SP198	SE Asia	Asia	China	39.428484	118.90827	SAMN17885320	Indoor
NA	SP212	SE Asia	Asia	China	35.40409	116.59243	SAMN17885333	Indoor

SE Asia: Southeast Asia.

Note: Modified table from Wang et al. (2024).

Table S2. MRM-settings and retention times of a putative chlorogenic acid isomer.

	RT [min]	Q1 [m/z]	Q3 [m/z]	Dwell time [ms]	CE [V]	Q1/Q3 Pre Bias [V]
Putative chlorogenic acid isomer	3,240	(+)355,10	163,15	32	-15	-23 / -17
		(+)355,10	135,10	32	-35	-23 / -14

RT: retention time.

CE: collision energy.

Qualifier is highlighted in grey.

Note: The analyte was added to the protocol in Method 1A of Malacrinò et al. (2024). Quantification was done relative to the isotope labelled phenylalanine standard in the original method (Schäfer et al., 2016). Due to the lack of the correct standard no correction factor was applied, and the data represent arbitrary units.

Table S3. Copper excess treatment effects *Spirodela polyrhiza* surface area growth rate independently of the outdoor pre-treatment.

	Chisq	Df	Pr(>Chisq)
Pre-treatment	0.4753	1	0.86
Treatment	159.7881	3	$4x10^{-11}$
Pre-treatment x Treatment	3.7389	3	0.74

Note: ANOVA test performed on a mixed effects model.

Spirodela polyrhiza genotype SP004.

Supporting methods

Additional information in experimental setup and statistical analyses.

Methods S1. Variables and models used to characterize the effects that the pre-treatment has on *Spirodela polyrhiza* fitness and morphology.

To assess the effects of the pre-treatment, the genotype and the treatment on plant fitness and morphology of six different genotypes, we included in the statistical analysis the following random variables:

Replicate Lineages started with a single individual (N = 6 per genotype) which offspring was divided into the copper and control pre-treatment, and afterwards, into control, copper, and aphid treatments (Figure **S2.1c**). Therefore, individuals coming from the same ancestor (Replicate in 2_SterileExp.xlsx) were not independent and were clustered with this variable.

Genotype Genetic cluster to which the samples belonged to (Genotype in 2_SterileExp.xlsx).

Offspring Despite starting fitness assays with plants at similar developmental stages, some plants showed no, one or two developmental pockets. As the presence of developmental pockets could create bias in fitness and morphology measurements based on surface area, but mostly on frond number, we included this information (offsringStart in 2_SterileExp.xlsx) into the analyses.

Rack Each rack allowed eight tubes to stay together during the whole propagation term, which was approximately 2.5 months. This means that four different genotypes with their two corresponding pre-treatments were always one next to the other. Therefore, this physical structure (Rack in 2_SterileExp.xlsx) was considered.

Branching The giant duckweed produces one offspring after the other, and the effects of using the first instead of the second offspring for fitness assays are unknown. Therefore, the information of being the first and second offspring when starting fitness assays (Figure **S2.1b**, G9:G10:G11 in 2_SterileExp.xlsx) was considered.

We excluded some of the random variables for specific questions when: first, the random factors did not explain any variance for most of the analysed response variables; second, when the fitted values deviated from the observed values – revised through plots; third, the random factor lost biological meaning. Thus, when using the pre-treatment ratios, the random variable *Offspring* lost biological meaning and was excluded from the statistical analyses.

Considering these random variables and the replicates from all six genotypes, we performed six different analyses:

1. To assess the effect of the pre-treatment on fitness and morphology ("Response" variable) under different environments, we used the model:
   Response ~Pre-treatment*Treatment
   +(1|Replicate)+(1|Genotype)+(1|Offspring)+(1|Rack)+(1|Branching).
2. To analyse the effect of the genotype on the pre-treatment ratios of fitness and morphology ("Pre-treatment ratio") under different treatments, we used the model:
   Pre-treatment ratio ~Genotype*Treatment+(1|Replicate)+(1|Branching).
3. To test the effect of the genotype on the absolute deviation of the pre-treatment ratios from null under different treatments, we used the model:
   Absolute deviation ~Genotype*Treatment+(1|Replicate).
4. To correlate the pre-treatment ratio of surface area growth rates among the three treatments, we first merged the data, keeping only samples present in all treatments. With this dataset, we applied the model
   pretreatment ratios in the treatment1 ~ pretreatment ratios in treatment2+(1|Genotype), and verified the R^2 values with simple linear models.
5. To correlate the different morphologies, we used the model:
   Morphology1 ~Morphology2*Treatment+(1|Pre-treatment)+(1|Genotype).
6. To correlate among the pre-treatment ratios of the morphologies we used the model
   Pre-treatment ratio1 ~Pre-treatment ratio2+(1|Genotype). We used the same model to correlate the pre-treatment ratios of morphology with the pre-treatment ratios of fitness within each treatment.

Methods S2. Variables and models used to characterize the effect of the pre-treatment on *Spirodela polyrhiza* metabolites.

To assess the effects of the pre-treatment and the treatment on the metabolite concentration of six genotypes of *S. polyrhiza*, we included in the statistical analysis the following random variables:

Replicate Lineages started with a single individual (N = 6 per genotype) which offspring was divided into the copper and control pre-treatment, and afterwards, into control, copper, and aphid treatments (Figure **S2.1**). Therefore, individuals coming from the same ancestor (Replicate in 2_SterileExp.xlsx) were not independent and thus clustered with this variable.

Genotype Genetic cluster to which the samples belonged to (Genotype in 2_SterileExp.xlsx).

Plate The samples were divided into four 96-well-plates, which were extracted and measure in different days. To avoid structuring, we organized samples by the number of replicate (one to six). Thus, plate one started with Replicate 1 of all genotypes and each of them with their pre-treatments and treatments. To consider the pure effect of the plate number, we introduced this variable (Platte in 2_SterileExp.xlsx) into the analysis.

Batch Within each plate, we worked with eight vertical wells per time, which included the addition of buffers during the extraction, as well as the reading of the metabolite concentrations. Therefore, we included the batch of 8 wells (Platte:ColumnInPlatte in 2_SterileExp.xlsx) into the analysis.

We excluded some of the random variables for specific models when: first, the random factors did not explain any variance for most of the analysed metabolites; second, when the fitted values visually deviated from the observed values.

Besides the screening of transgenerationally plastic metabolites within each treatment, we performed four additional analyses considering the replicates of all six *S. polyrhiza* genotypes:

1. To assess the effect of both, the pre-treatment and the treatment on those transgenerationally plastic metabolites (i.e. cyanidins, OPDA and tryptamine), we used the model:
   Concentration ~Pre-treatment*Treatment+(1|Genotype)+(1|Replicate)+(1|Plate)+(1|Batch).
   Furthermore, to assess the metabolite concentration differences within each treatment, we used the model:
   Concentration ~Pre-treatment+(1|Genotype)+(1|Replicate)+(1|Plate)+(1|Batch).
2. To assess the effects of the pre-treatment and genotype on the concentrations of jasmonates within the aphid herbivory treatment, we used the model:

Concentration ~Pre-treatment*Genotype+(1|Replicate)+(1|Plate)+(1|Batch).

3. To additionally identify the effect of the genotype on all metabolites, we used the model: Concentration ~Pre-treatment*Treatment*Genotype+(1|Replicate)+(1|Batch).
4. To assess whether the metabolites that accumulated higher concentrations upon copper pre-treatment – namely anthocyanins, tryptamine and jasmonates – were induced by first-time copper exposure, we focused on control pre-treated plants that were challenged to control, copper and aphid treatments. Thereto, to analyse the induction changes under first time stress exposure we used the model:
   Concentration ~Treatment+(1|Genotype)+(1|Replicate)+(1|Plate)+(1|Batch).

Methods S3. Assessing the effects of *Spirodela polyrhiza* induced jasmonates in the growth rates of *Rhopalosiphum nymphaeae*.

To assess the reproducibility of aphid growth rates in plants pre-treated with methyl jasmonate, we cultivated six plant replicates of *S. polyrhiza* genotype SP050, each consisting of two pseudo-replicates which included 10 fronds in different developmental stages – three to four fronds per colony. We placed the pseudo-replicates in 7.5 mL of 100 µM methyl jasmonate and control conditions within 6-well-plates ("Induction"). Methyl jasmonate was dissolved in N-medium by shaking at 1200 rpm for 2 hours until no globules of fat were visible. Plates were sealed with parafilm for 96 hrs, after which, a total of 20 fronds per replicate were moved into 150 mL N-medium with and without 10 aphids per replicate. All remaining fronds from the induction phase were flash-frozen and stored in -80°C for posterior metabolite extraction. This experiment was performed under 26°C, 135 µM photons m^2 s^{-1}, 16:8 hours light/dark.

To assess whether the application of methyl jasmonate alters OPDA and jasmonates, we extracted and measured these metabolites as described in "*Effects of copper pre-treatment on* Spirodela polyrhiza *metabolites*" within Materials and Methods. Afterwards, to test whether methyl jasmonate induced the levels of these metabolites, we used the model OPDA ~Pretreat+(1|Plate).

Besides the effects of methyl jasmonates on jasmonate induction, we measured the effects of methyl jasmonate on aphid growth rates. Thereto, we used the model
Aphid growth rate ~Induction+(1|Plate)).

Within this experiment, *Plate* considered the physical structure during the induction phase.

Methods S4. Variables to assess the effects of methyl jasmonate on *Rhopalosiphum nymphaeae* growth rates.

To assess the effects of methyl jasmonate on the aphid *R. nymphaeae* growth rates, we included in the statistical analysis the following random variables:

Replicate During the pre-treatment phase ("induction"), replicates contained three fronds per tube which were placed into the different treatments. Therefore, these fronds were not independent (Replicate in 4_InductionExp.xlsx) and thus clustered with this variable.

Group Due to the total number of samples (N = 72), we divided the experiment into three batches of four replicates, each performed one day after the other. Therefore, we introduced this variable (DateStart in 4_InductionExp.xlsx) to consider the structure of time.

Equations used for the transformation of data

Equation S1. Relative growth rate (Hunt, 1982).

$$\frac{\ln(\text{final measurement}) - \ln(\text{initial measurement})}{\text{number of days}}$$

Equation S2. Pre-treatment ratio (Huber et al., 2021).

$$\frac{\text{plant fitness or phenotype of copper pre} - \text{treated plants}}{\text{mean (plant fitness or phenotype of control pre} - \text{treated plants)}}$$

and

$$\frac{\text{aphid fitness of individuals growing on copper pre} - \text{treated plants}}{\text{mean (aphid fitness of individuals growing on control pre} - \text{treated plants)}}$$

References

Huber, M., Gablenz, S. & Höfer, M. (2021) Transgenerational non-genetic inheritance has fitness costs and benefits under recurring stress in the clonal duckweed *Spirodela polyrhiza*. *Proceedings of the Royal Society B: Biological Sciences.* 288(1955): 20211269. doi: https://doi.org/10.1098/rspb.2021.1269.

Hunt, R. (1982) *Plant growth curves. the functional approach to plant growth analysis.* London, UK, Edward Arnold Ltd.

Malacrinò, A., Böttner, L., Nouere, S., Huber, M., Schäfer, M. & Xu, S. (2024) Induced responses contribute to rapid adaptation of *Spirodela polyrhiza* to herbivory by *Lymnaea stagnalis*. *Communications Biology.* 7, 81. doi: https://doi.org/10.1038/s42003-023-05706-0.

Schäfer, M., Brütting, C., Baldwin, I. T. & Kallenbach, M. (2016) High-throughput quantification of more than 100 primary- and secondary-metabolites, and phytohormones by a single solid-phase extraction based sample preparation with analysis by UHPLC–HESI–MS/MS. *Plant Methods.* 12, 30. doi: https://doi.org/10.1186/s13007-016-0130-x.

Wang, Y., Duchen, P., Chávez, A., Sree, K. S., Appenroth, K. J., Zhao, H., Höfer, M., Huber, M. & Xu, S. (2024) Population genomics and epigenomics of *Spirodela polyrhiza* provide insights into the evolution of facultative asexuality. *Communications Biology.* 7, 581. doi: https://doi.org/10.1038/s42003-024-06266-7.
